# Socioeconomic inequalities in diagnostics, care and survival outcomes for hepatocellular carcinoma in Sweden: a nationwide cohort study

**DOI:** 10.1016/j.lanepe.2025.101273

**Published:** 2025-03-20

**Authors:** Juan Vaz, Hannes Hagström, Malin Sternby Eilard, Magnus Rizell, Ulf Strömberg

**Affiliations:** a School of Public Health and Community Medicine, Institute of Medicine, Sahlgrenska Academy, University of Gothenburg, Sweden; b Department of Medicine, Huddinge, Karolinska Institutet, Stockholm, Sweden; c Department of Medicine, Halland Hospital Halmstad, Halmstad, Sweden; d Unit of Hepatology, Department of Upper GI Diseases, Karolinska University Hospital, Stockholm, Sweden; e Department of Surgery, Institute of Clinical Sciences, Sahlgrenska Academy, University of Gothenburg, Gothenburg, Sweden; f Department of Transplantation, Sahlgrenska University Hospital, Region Västra Götaland, Gothenburg, Sweden

**Keywords:** Hepatocellular carcinoma, Socioeconomic status, Inequalities, Surveillance, Survival

## Abstract

**Background:**

Hepatocellular carcinoma (HCC) is a leading cause of cancer-related mortality worldwide. This study evaluates how strongly socioeconomic factors associate with diagnostics, treatment, and survival among patients with HCC in Sweden.

**Methods:**

All adult patients registered with a diagnosis of HCC in the Swedish quality register for liver cancer between 2011 and 2021 were included. Household income was classified as low (first quartile; poorest), medium (second or third quartile), or high (in fourth quartile; wealthiest) based on the overall distribution of household income across all household in Sweden. Outcomes included likelihood of diagnosis under surveillance, early-stage diagnosis (Barcelona Clinic Liver Cancer [BCLC] staging 0-A), and receipt of curative treatment (ablation, resection or liver transplantation), as well as mortality risk.

**Findings:**

Among 5490 patients, a significant association was found between low household income and decreased likelihood of diagnosis while under surveillance (adjusted odds ratio [aOR] 0·63; 95% confidence interval [CI]: 0·50–0·80), early-stage diagnosis (aOR 0·58; 95% CI: 0·51–0·67), and curative treatment receipt (aOR 0·65; 95% CI: 0·50–0·85). After adjustments for all variables in the BCLC, other sociodemographic variables, comorbidities, and cirrhosis status, patients with low household income had an adjusted hazard ratio for mortality of 1·29 (95% CI: 1·15–1·45) compared to patients with high household income.

**Interpretation:**

Socioeconomic disparities associate markedly with more advanced stage at HCC diagnosis, less curative treatment, and poorer survival in Sweden. Addressing these disparities through targeted public health interventions may improve HCC care and outcomes in socioeconomically disadvantaged populations.

**Funding:**

The 10.13039/501100002794Swedish Cancer Society—10.13039/501100002794Cancerfonden.

**Research in context**

**Evidence before this study**

We initially searched PubMed from its inception to June 2024 using keywords such as “liver cancer”, “hepatocellular carcinoma [HCC]”, “hepatoma”, “socioeconomic status”, “socioeconomic position”, “deprivation”, “disparity”, “inequity”, and “inequality”. Two systematic reviews published in 2024 reported significant racial and ethnic disparities in HCC treatment receipt in the United States. A comprehensive review published in April 2024 concluded that existing research indicates an association between lower socioeconomic status and delayed cancer diagnosis, reduced access to optimal treatment, and poorer outcomes in Europe, particularly among individuals living in economically disadvantaged areas. However, nationwide studies specifically examining HCC in high-income settings, such as Sweden, are limited.

**Added value of this study**

By examining data from national registers including the quality register for HCC with granular data, this study sheds light on how socioeconomic inequities persist even within a system designed to ensure equitable healthcare access. Our findings highlight the gaps in achieving healthcare equity, particularly in diagnostics and treatment stages, despite universal coverage. This study also quantifies these disparities, providing a valuable foundation for future efforts to target and mitigate socioeconomic barriers in HCC care.

**Implications of all the available evidence**

Current evidence suggests that while universal healthcare reduces financial barriers, it may not fully eliminate socioeconomic inequities in HCC diagnosis, treatment, and survival. These findings highlight the need for targeted interventions within universal healthcare systems to address remaining inequities associated with socioeconomic status, particularly along care pathways. Future research should focus on identifying specific factors within healthcare delivery that contribute to these disparities and assessing interventions that may improve healthcare equity for HCC and other cancer patients in universal healthcare contexts.

## Introduction

Hepatocellular carcinoma (HCC) is the most common primary liver cancer and a leading cause of cancer-related mortality globally.^1^ Compelling evidence identifies liver cirrhosis, hepatitis B (HBV) and C (HCV) infections, and metabolic disorders as key biological drivers of HCC incidence.^2^ Increasingly, there is also recognition of the influence of socioeconomic factors on HCC incidence, diagnosis, treatment, and survival outcomes.^3^ Lower socioeconomic status (SES) has been linked to increased HCC incidence and poorer outcomes.^3^^,^^4^ These inequalities often intersect with other risk factors, including alcohol use, smoking, and healthcare access, creating a complex landscape of HCC burden shaped not only by biological but also by individual and contextual socioeconomic factors.^3^

While there is no direct causal pathway between SES and HCC development, low SES is consistently associated with key HCC risk factors, including HCV, alcohol-related liver disease (ALD), and obesity, all of which are more prevalent in disadvantaged populations.^5^, ^6^, ^7^ Intravenous drug use, a major transmission route for HBV and HCV in many European countries,^8^ is more common in low-income groups, further exacerbating the risk of cirrhosis and HCC in these populations.^9^ Additionally, low SES is linked to non-causal factors, such as health literacy and access to care.^10^ For example, delayed diagnosis of cirrhosis and HCC is more common among lower SES groups, often resulting in late-stage presentation when curative treatment is less feasible.^11^

With a high-quality, tax-funded healthcare system accessible to all residents and broad social safety nets, Sweden offers a unique setting to assess associations between SES and HCC outcomes, as it reduces the financial barriers to healthcare access seen in many other countries. Recent studies indicate that HCC is one of the most socioeconomically polarised cancers in Sweden, showing strong associations between lower household income, higher neighbourhood deprivation, and increased HCC incidence.^12^ Immigrants from non-Nordic countries also have higher HCC rates than those born in Nordic countries, even after adjustment for SES.^12^ This aligns with broader European trends, where ethnic minorities and immigrant populations face greater socioeconomic deprivation and health disparities, contributing to elevated cancer risks.^3^

Despite this, nationwide studies specifically examining socioeconomic inequalities in HCC care within high-income, universal healthcare systems like Sweden's are limited. Moreover, knowledge about the specific social determinants most associated with disparities in HCC care remains scarce. Building on these insights, our study aims to identify and quantify associations between various sociodemographic factors and three critical aspects of HCC care: early diagnosis, treatment, and survival.

## Methods

### Study population

We utilized data from the Swedish quality register for liver cancer (SweLiv), cross-linked with data from several other national health care registers and demographic databases, using the unique personal identification number assigned to all permanent residents of Sweden.^13^ SweLiv was validated against the National Cancer Register in 2014, and currently covers over 95% of all documented cases of liver cancer in the country.^13^

We included all patients aged 18 years or older who were registered in SweLiv with a diagnosis of HCC (International Classification of Diseases 10th Edition [ICD-10] code C22.0) between January 1, 2011, and December 31, 2021. The start date was chosen to allow for the evaluation of national HCC guidelines introduced in late 2012, which previously have been linked to an increase in the proportion of treatments aimed at cure.^13^ The end of the study period was selected to ensure a minimum follow-up time of two years for patients diagnosed in 2021 who remained alive in May 23, 2024, which was the end of the follow-up period.

### Sociodemographic characteristics

Sociodemographic data at the time of HCC diagnosis were provided by Statistics Sweden. Country of birth was categorized as “Nordic” (born in Sweden, Norway, Denmark, Finland, or Iceland) or “non-Nordic” (all other countries). Marital status was categorised as married, single, divorced/separated, or widowed. SES was assessed at both individual and neighbourhood levels.^12^ Household income was selected as the main individual-level SES indicator, as educational level, is more frequently missing for immigrants.^14^ Additionally, household income provides more complete data and may, arguably, better represent individual SES; e.g., those with low educational attainment belonging to high-income households are classified as having high SES. However, educational level was included as a secondary SES indicator, given its frequent use in prior research. Educational level was defined based on years of formal education: low (≤9 years), medium (10–12 years), and high (>12 years).

Household income was defined as disposable income per household per consumption unit, including all taxable and tax-exempt income, minus taxes and negative transfers, adjusted for household composition Statistics Sweden's weighting. Each patient's household income was classified as low (first quartile; poorest), medium (second or third quartile), or high (in fourth quartile; wealthiest) based on the overall distribution of household income across all household in Sweden.^14^

Residential area at the time of diagnosis and neighbourhood-level socioeconomic data were obtained from Statistics Sweden's Demographic Statistical Areas (DeSO), introduced in 2018 to track segregation and socioeconomic conditions. In 2018, there were 5985 DeSOs across Sweden, with populations ranging from 600 to 4300 individual (median: 1600).^15^ The populations for each DeSO, including proportions of immigrants and low income residents, are sensitive to demographic shifts and immigration; thus, we collected data for each year of the study period.

For each DeSO, year-specific proportions of residents with low household income were calculated, ranking DeSOs from least to most deprived. Based on this ranking, DeSOs were divided into national quintiles (Q1–Q5), with each quintile containing about 1197 DeSOs, from least deprived (Q1) to most deprived (Q5). Each patient's neighbourhood-level deprivation, as a measure of contextual SES, was determined by their residential DeSO at the time of HCC diagnosis.^15^

### Diagnostic pathways, liver diseases, and comorbidity

Data on diagnostic pathways, presence of cirrhosis and the underlying aetiology (defined below), staging, and treatments were retrieved from SweLiv. As SweLiv has not yet undergone validation for data on cirrhosis and underlying liver disease, additional patient data were obtained from the validated National Patient Register (NPR).

Diagnostic pathways are recorded in SweLiv for each patient at the time of registration. Patients were classified as diagnosed through surveillance based on a predefined variable in SweLiv, which is completed by the reporting health professional. SweLiv categorises diagnostic pathways into three groups: surveillance, clinical symptoms (symptomatic diagnosis by intent), incidental diagnosis (unexpected tumour detection via radiology or surgery). The classification of a patient as diagnosed through surveillance is based on physician-reported data at multidisciplinary team cancer conferences and not from an algorithmic determination from clinical characteristics. SweLiv does not provide information on the specific surveillance modality used, adherence to surveillance protocols, or frequency of surveillance examinations.

HCC surveillance protocols in Sweden aligns with the European Association for the Study of the Liver guidelines,^16^ which recommend semi-annual (every 6 months) ultrasound screening without the use of biomarkers (such as alpha-fetoprotein), for all eligible patients with liver cirrhosis regardless of aetiology, and for high-risk individuals with chronic HBV or acute intermittent porphyria even in the absence of cirrhosis. Surveillance is primarily conducted in specialist hepatology clinics but may also be coordinated through general gastroenterology, infectious disease, internal medicine or primary care departments. Despite these, prior studies from Sweden indicate that a significant number of eligible patients are not surveilled according to guidelines,^17^ which also has been shown by a meta-analysis.^18^

Aetiologies of underlying liver disease were obtained from SweLiv or determined using data from the NPR. We defined seven distinct aetiologies: HBV, HCV, ALD, metabolic dysfunction-associated steatotic liver disease (MASLD), “other liver diseases”, which encompasses rare liver conditions such as primary biliary cholangitis and autoimmune hepatitis; cryptogenic cirrhosis, and “no diagnosed liver disease”. As with many other cancer types, the causes of HCC are multifactorial, which must be considered. Consistent with previous Swedish studies,^11^ we employed the following ranking system (from highest to lowest) for patients with more than one potential cause of HCC: rare liver diseases, HBV, HCV, ALD, MASLD, cryptogenic cirrhosis, and “no diagnosed liver disease”.

Cirrhosis status was determined using data from SweLiv and the NPR, as previously described.^11^ The ICD codes used to identify cirrhosis in the NPR have been validated, showing a high positive predictive value (>90%).^19^ Liver cirrhosis severity was assessed using the Child-Pugh score. Patients with cirrhosis were classified as having decompensated cirrhosis if the Child Pugh score was >7. Comorbidities, included as covariates in the regression models described below, were selected based on their clinical relevance for HCC prognosis and treatment decisions and were identified using data from the NPR or the Prescribed Drug Register (Table S1).

### Staging and treatment

The Swedish treatment algorithm for HCC in effect during the study period is provided as Supplementary data (Figure S1). While this algorithm is based on the BCLC system,^20^ there are notable differences that require clarification. First, the Swedish algorithm categorizes HCC into four stages: early (BCLC 0 and A), intermediate (BCLC B), advanced (BCLC C), and terminal (BCLC D). Second, unlike the BCLC, the Swedish algorithm evaluates performance status by considering all symptoms, not just those related to HCC. Third, some patients may be recommended for curative treatment options other than transplantation, even if they have impaired liver function or an ECOG score ≥2.

Treatment data were obtained from SweLiv and classified as curative if ablation, resection, or transplantation were registered. When multiple curative treatments were administrated, transplantation was prioritised as the primary treatment (regardless of other treatments), followed by resection if performed before ablation, and ablation if performed before resection. Transarterial chemoembolisation, systemic therapy, or radiotherapy were considered palliative treatments. Patients for whom no antitumour treatment was identified were classified as receiving best supportive care. Curative treatments received were verified by cross-referencing procedural codes in the NPR for validation, and cases registered in SweLiv but not in the NPR were excluded from analysis.

### Outcomes

We assessed the associations between individual- and neighbourhood-level sociodemographic factors across three key aspects of HCC care: diagnosis, treatment, and survival. The main outcomes were the likelihood of i) HCC diagnosis during surveillance, ii) early-stage HCC diagnosis, iii) receiving curative treatment; and iv) the risk of mortality.

### Statistical analysis

Continuous variables were reported as medians with interquartile ranges (IQRs), and categorical variables as counts and percentages. Group differences were assessed using chi-square (χ^2^) test for categorical variables and the Mann–Whitney *U* test for continuous variables.

Univariable and multivariable logistic regression models estimated odds ratios (ORs) and adjusted odds ratios (aORs) for the binary outcomes. For the likelihood of diagnosis via surveillance, only patients with cirrhosis were included, as they are the primary target group. ORs for individual-level SES were adjusted for neighbourhood-level SES, and vice versa. The final model adjusted SES associations for age, sex, country of birth, marital status, period of diagnosis, and aetiology.

For early-stage HCC diagnosis, all patients were included, with adjustments for the same variables as above, plus cirrhosis status (no cirrhosis, compensated, decompensated). The likelihood of curative treatment was adjusted for age, sex, country of birth, marital status, period of diagnosis, aetiology, cirrhosis status, BCLC parameters (tumour size, number, tumour thrombosis, metastasis, ECOG) and comorbidities.

To examine whether the effect of individual SES on HCC outcomes varied depending on the socioeconomic characteristics of the residential area, we tested interactions between individual-level SES variables, and neighbourhood deprivation, as well as country of birth and marital status.

Kaplan–Meier estimates with Greenwood confidence intervals (CI) determined median survival and survival probabilities across sociodemographic groups. Cox regression models provided unadjusted and adjusted hazard ratios (HRs and aHRs) for mortality. Final Cox models were adjusted for all variables used in logistic regression model for curative treatment. To assess the proportional hazards assumption, we tested Schoenfeld residuals using *estat phtest*. Additionally, we considered log–log survival plots.

The study period was divided into three groups (2011–2014, 2015–2019, and 2020–2021) to assess changes over time in HCC aetiologies and outcomes. The first period accounts for potential delays in the implementation of national guidelines introduced in late 2012, while 2020–2021 reflects the impact of the COVID-19 pandemic, which has been associated with reduced patient surveillance in some countries.^21^^,^^22^ Trends in HCC aetiologies between periods were assessed by the Mantel–Haenszel test, and the likelihoods of outcomes and mortality risks across periods were analysed using “period of diagnosis” as a categorical variable, with 2011–2014 as the reference.

Patients diagnosed with HCC who have low SES tend to present with worse performance status at diagnosis compared to higher SES groups,^11^ which can impact all subsequent outcomes. Sensitivity analyses, excluding the ECOG covariate, were conducted to estimate aORs and aHRs for all outcomes across household income groups, stratified by ECOG scores (0–1 and ≥2).

### Handling of missing data

Missing data were primarily related to ECOG, which was uncertain in 877 patients (16%). A detailed review of the dataset showed that only 350 of these patients (6% of the total cohort) received either curative (n = 18) or palliative (n = 332) treatment. Since curative and palliative treatments are generally administered to patients with ECOG ≤1, we assigned ECOG 0 to patients receiving curative treatment, those without comorbidities, and those with compensated cirrhosis, and ECOG 1 to the remaining patients who received palliative treatment. For the 527 uncertain ECOG cases remaining, all of whom received best supportive care, we classified them as ECOG ≥2, aligning with clinical expectations and treatment patterns. Since missing data for other variables (educational level, diagnostic pathways, and number of tumours) were low (2%–4%), we opted to use complete case analysis as our primary approach in multivariable models.

### Ethics approval

This study was approved by the Central Ethical Review Board in Sweden (decision number 2023-04555-01 and 2024-05174-02). Due to the retrospective nature of this study, patient consent was not required.

### Role of the funding source

The funder has no role in study design, data collection, data analysis, data interpretation, preparation of the report, or decision to publish.

## Results

### Patient characteristics

A total of 5490 patients with HCC were identified. The median age at diagnosis was 70 years (IQR 62–76, range 18–97), with the majority being male (76%). Nearly half of the cohort had low household income (46%), while only 13% had high household income. MASLD was the most common cause of HCC (27%), followed by HCV (25%). Patient characteristics by household income are shown in Table 1, with stratifications by other sociodemographic factors and by cirrhosis status are provided in the Supplementary data (Tables S2–S7).

**Table 1:** Baseline characteristics of 5490 patients diagnosed with hepatocellular carcinoma in Sweden between 2011 and 2021.

	Household income level			
	Low	Medium	High	Total
	2546 (46)	2218 (41)	726 (13)	5490 (100)
**Male sex**	1871 (73)	1736 (78)	572 (79)	4179 (76)
**Median age**	70 (62–77)	71 (63–76)	68 (62–73)	70 (62–76)
**Country of birth**				
Nordic	2077 (82)	1953 (88)	664 (91)	4694 (85)
Non-Nordic	469 (18)	265 (12)	62 (9)	796 (15)
**Marital status**				
Married	805 (32)	1215 (55)	493 (68)	2513 (46)
Single	669 (26)	344 (15)	83 (11)	1096 (20)
Divorced/separated	688 (27)	438 (20)	101 (14)	1227 (22)
Widowed	384 (15)	221 (10)	49 (7)	654 (12)
**Educational level**				
High (>12 years)	191 (7)	406 (18)	298 (41)	895 (16)
Medium (10–12 years)	964 (38)	1127 (51)	300 (41)	2391 (44)
Low (≤9 years)	1297 (51)	660 (30)	123 (17)	2080 (38)
Unknown	94 (4)	25 (1)	5 (1)	124 (2)
**Neighbourhood deprivation**				
Q1 (least)	177 (7)	317 (14)	208 (29)	702 (13)
Q2	332 (13)	440 (20)	172 (24)	944 (17)
Q3	464 (18)	475 (21)	54 (21)	1093 (20)
Q4	624 (25)	507 (23)	109 (15)	1240 (23)
Q5 (most)	949 (37)	479 (22)	83 (11)	1498 (27)
**Aetiology**				
Hepatitis B	273 (11)	133 (6)	34 (5)	440 (8)
Hepatitis C	777 (31)	445 (20)	138 (19)	1360 (25)
ALD	362 (14)	472 (21)	196 (27)	1030 (19)
MASLD	630 (25)	666 (30)	171 (23)	1467 (27)
Other liver diseases	127 (5)	165 (8)	71 (10)	363 (6)
Cryptogenic cirrhosis	154 (6)	127 (6)	60 (8)	341 (6)
No diag. liver disease	223 (8)	210 (9)	56 (8)	489 (9)
**Liver cirrhosis**	1958 (77)	1635 (74)	571 (79)	4164 (76)
Child-Pugh ≤7^a^	1452 (74)	1294 (79)	439 (77)	3185 (76)
Child-Pugh >7^a^	506 (26)	341 (21)	132 (23)	979 (24)
**Diagnostic pathway**				
Surveillance	593 (23)	590 (27)	218 (30)	1401 (26)
Clinical symptoms	1368 (54)	1156 (52)	356 (49)	2880 (52)
Incidental finding	471 (19)	387 (17)	131 (18)	981 (18)
Missing	114 (4)	85 (4)	21 (3)	220 (4)
**ECOG PS**				
0	584 (23)	770 (35)	315 (43)	1669 (30)
1	768 (30)	709 (32)	214 (30)	1691 (31)
≥2	1194 (47)	739 (33)	197 (27)	2130 (39)
**Tumour size** (**mm)**				
Median	48 (26–85)	43 (25–80)	40 (22–80)	45 (25–80)
≤20	395 (16)	403 (18)	144 (20)	942 (17)
21–29	360 (14)	342 (16)	120 (16)	822 (15)
≥30	1791 (70)	1473 (66)	462 (64)	3726 (68)
**Number of tumours**				
1	1347 (53)	1241 (56)	389 (54)	2977 (54)
2–3	555 (22)	512 (23)	184 (25)	1251 (23)
>3	557 (22)	419 (19)	137 (19)	1113 (20)
Uncertain	87 (3)	46 (2)	16 (2)	149 (3)
**Lymph node metastasis**	324 (13)	214 (10)	72 (10)	610 (11)
**Extrahepatic metastasis**	439 (17)	266 (12)	94 (13)	799 (15)
**Tumour thrombosis**	387 (15)	243 (11)	78 (11)	708 (13)
**Stage at diagnosis**				
Early	716 (28)	879 (40)	310 (43)	1905 (35)
Intermediate	505 (20)	477 (21)	156 (21)	1138 (21)
Advanced	286 (11)	285 (13)	120 (17)	691 (12)
Terminal	1039 (41)	577 (26)	140 (19)	1756 (32)
**Comorbidities**				
Arterial hypertension	948 (37)	832 (38)	255 (35)	2035 (37)
Type 2 diabetes	1049 (41)	999 (45)	306 (42)	2354 (43)
Coronary artery disease	489 (19)	421 (19)	98 (13)	1008 (18)
Cerebrovascular disease	301 (12)	222 (10)	69 (10)	592 (11)
Chronic kidney disease	208 (8)	184 (8)	53 (7)	443 (8)
COPD	406 (16)	265 (12)	52 (7)	723 (13)
**Treatment**				
Transplantation	123 (5)	148 (7)	79 (11)	350 (6)
Resection	304 (12)	416 (19)	136 (19)	856 (15)
Ablation	358 (14)	369 (16)	132 (18)	859 (16)
Palliative	682 (27)	673 (30)	216 (30)	1571 (29)
Best supportive care	1079 (42)	612 (28)	163 (22)	1854 (34)

ALD: alcohol-related liver disease; COPD: chronic obstructive pulmonary disease; ECOG PS: Eastern Cooperative Oncology Group performance status; MASLD: metabolic dysfunction-associated steatotic liver disease.

a Percentage of patients with cirrhosis.

As previously described, interactions between individual-level variables and neighbourhood deprivation were tested. Given the lack of significant interactions, we proceeded with main effects models for SES variables in our multivariable analyses.

### Surveillance

Among patients with cirrhosis (n = 4164), 1387 (33%) were diagnosed with HCC while under surveillance. The proportion of patients with cirrhosis and HCC diagnosis through surveillance increased with household income: 30% at low, 36% at medium, and 38% at high-income level (p < 0.001).

**Fig. 1:**
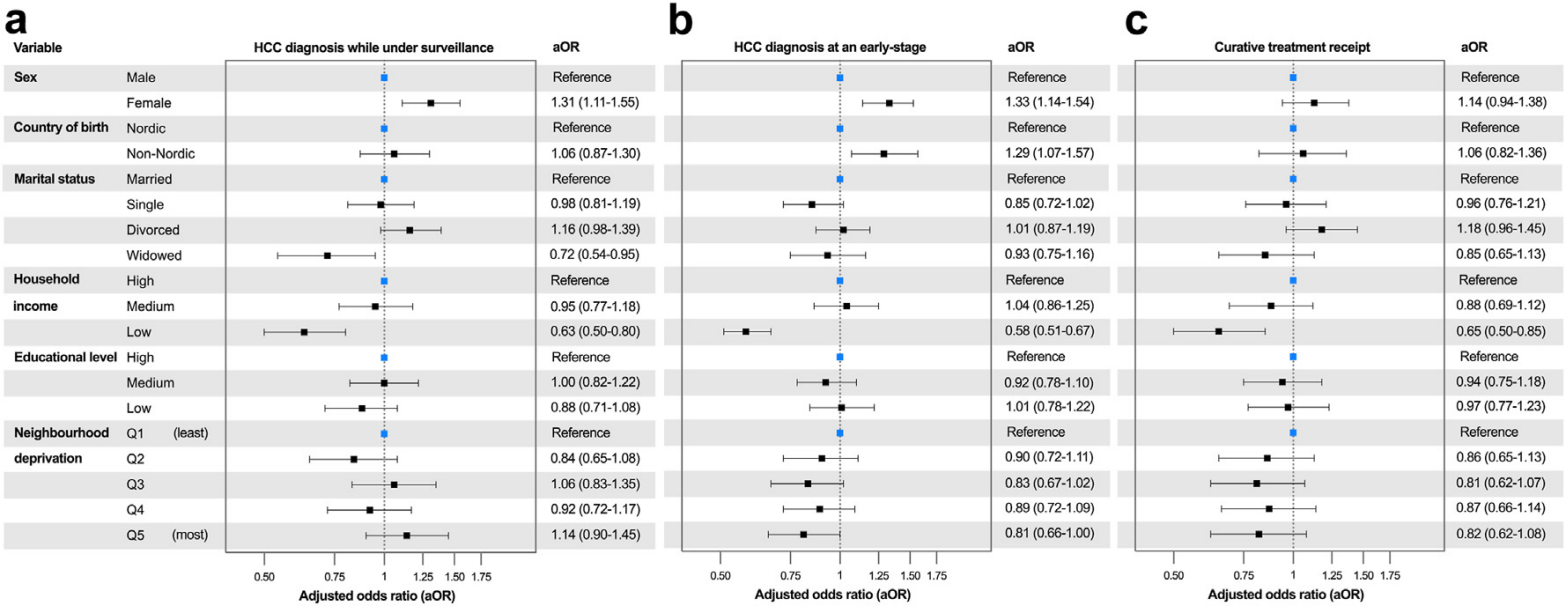
Results from multivariable logistic regression models showing the likelihood of: (a) hepatocellular carcinoma (HCC) diagnosis during surveillance, (b) early-stage HCC diagnosis, and (c) receiving curative treatment in Sweden from 2011 to 2021. The multivariable model in panel (a) was adjusted for all variables shown, plus age, period of diagnosis (2011–2014, 2015–2019, 2020–2021), and aetiology and was limited to patients with cirrhosis (n = 4178). In panel (b), all patients (n = 5490) were included, with adjustments for the same variables as in (a), as well as cirrhosis status (no cirrhosis, compensated, decompensated). In panel (c), adjustments for the same variables as in (b) were done, as well as for tumour size, number, tumour thrombosis, metastasis, performance status, and comorbidities (arterial hypertension, type 2 diabetes, coronary artery disease, cerebrovascular disease, chronic kidney disease, chronic obstructive pulmonary disease).

In the fully adjusted logistic regression model, low household income was significantly associated with a decreased likelihood of diagnosis through surveillance, with an aOR of 0·63 (95% CI: 0·50–0·80) compared to high household income (Fig. 1a), and an aOR of 0·70 (95% CI: 0·60–0·82) compared to medium household income. Unadjusted and adjusted estimates for all included variables are presented in Table S8.

### Tumour stage at diagnosis

A total of 1905 patients (35%) were diagnosed with early-stage HCC. Patients with high and medium household incomes were more often diagnosed at early stages compared to those with low income (43% and 40% vs. 28%; p < 0·001), though no significant difference was found between high- and medium-income levels (p = 0·385).

In the fully adjusted logistic regression model, low household income was associated with a reduced likelihood of early-stage diagnosis compared to high household income (aOR 0·58; 95% CI: 0·51–0·67) (Fig. 1b), and with an aOR of 0·62 (95% CI: 0·55–0·69) compared to medium household income. Unadjusted and adjusted estimates for all included variables are presented in Table S9.

### Curative treatment receipt

A total of 2065 patients (38%) received curative treatment, with the highest proportion (48%) observed among patients with high household income (Table 1). After full adjustment in the logistic regression model, low household income was significantly associated with a lower likelihood of receiving curative treatment (aOR 0·65; 95% CI: 0·50–0·85) compared to high household income (Fig. 1c), and with an aOR of 0·78 (95% CI: 0·63–0·90) compared to medium household income. Unadjusted and adjusted estimates for all included variables are presented in Table S10.

### Survival

During a total follow-up of 13,786 person-years, 4398 patients (80%) died. The median survival time was 1·41 years (95% CI: 1·34–1·49), and the 1- and 5-year survival probabilities were 0·57 (95% CI: 0·56–0·58) and 0·23 (95% CI: 0·22–0·24), respectively. Patients with low household income had worse median survival and lower 1- and 5-year survival probabilities compared to higher income groups (Table 2).

**Table 2:** Survival probabilities of patients diagnosed with hepatocellular carcinoma in Sweden between 2011 and 2021.

	N	Deaths	Survival probability (95% CI)		Median survival in years (95% CI)
			1-year	5-year	
**Overall**	5490	4398	0.57 (0.56–0.58)	0.23 (0.22–0.24)	1.41 (1.34–1.49)
**Sex**					
Male	4179	3370	0.57 (0.55–0.58)	0.23 (0.21–0.24)	1.38 (1.30–1.47)
Female	1311	1028	0.59 (0.56–0.61)	0.25 (0.22–0.27)	1.49 (1.34–1.75)
**Country of birth**					
Nordic	4694	3833	0.57 (0.55–0.58)	0.22 (0.21–0.23)	1.37 (1.29–1.46)
Non-Nordic	796	565	0.60 (0.57–0.64)	0.31 (0.27–0.34)	1.71 (1.40–2.04)
**Marital status**					
Married	2513	1996	0.58 (0.56–0.60)	0.24 (0.22–0.26)	1.46 (1.35–1.57)
Single	1096	870	0.57 (0.54–0.60)	0.24 (0.21–0.26)	1.42 (1.23–1.57)
Divorced	1227	959	0.60 (0.57–0.63)	0.25 (0.23–0.28)	1.58 (1.41–1.76)
Widowed	654	573	0.50 (0.46–0.54)	0.14 (0.12–0.18)	0.99 (0.85–1.14)
**Household income**					
High	726	525	0.66 (0.63–0.70)	0.29 (0.26–0.33)	2.04 (1.70–2.43)
Medium	2218	1739	0.64 (0.62–0.66)	0.27 (0.25–0.28)	1.77 (1.65–1.93)
Low	2546	2134	0.48 (0.47–0.50)	0.18 (0.17–0.20)	0.92 (0.85–1.02)
**Educational level**					
High	895	677	0.61 (0.58–0.64)	0.29 (0.26–0.32)	1.63 (1.41–1.89)
Medium	2391	1887	0.60 (0.57–0.61)	0.24 (0.23–0.26)	1.56 (1.44–1.70)
Low	2080	1736	0.53 (0.51–0.56)	0.19 (0.17–0.21)	1.19 (1.07–1.30)
**Neighbourhood deprivation**					
Q1 (least)	702	562	0.60 (0.56–0.63)	0.23 (0.20–0.27)	1.60 (1.39–1.90)
Q2	944	739	0.58 (0.55–0.61)	0.25 (0.23–0.28)	1.41 (1.25–1.64)
Q3	1093	873	0.56 (0.53–0.59)	0.24 (0.21–0.26)	1.32 (1.14–1.44)
Q4	1240	1014	0.58 (0.55–0.61)	0.20 (0.18–0.23)	1.44 (1.28–1.57)
Q5 (most)	1511	1210	0.55 (0.53–0.58)	0.23 (0.21–0.25)	1.38 (1.22–1.54)
**Stage at diagnosis**					
Early	1905	1090	0.89 (0.88–0.91)	0.50 (0.47–0.52)	5.02 (4.60–5.40)
Intermediate	1138	919	0.76 (0.73–0.78)	0.23 (0.20–0.26)	2.24 (2.03–2.45)
Advanced	691	669	0.36 (0.32–0.39)	0.03 (0.02–0.04)	0.68 (0.62–0.72)
Terminal	1756	1720	0.19 (0.17–0.21)	0.02 (0.01–0.03)	0.28 (0.26–0.30)
**Curative treatment**					
Transplantation	260	64	0.98 (0.96–0.99)	0.83 (0.78–0.87)	–
Resection	883	474	0.92 (0.90–0.93)	0.55 (0.52–0.59)	6.04 (5.25–6.97)
Ablation	922	572	0.92 (0.90–0.93)	0.44 (0.41–0.48)	4.35 (4.02–4.80)

CI: confidence interval.

**Fig. 2:**
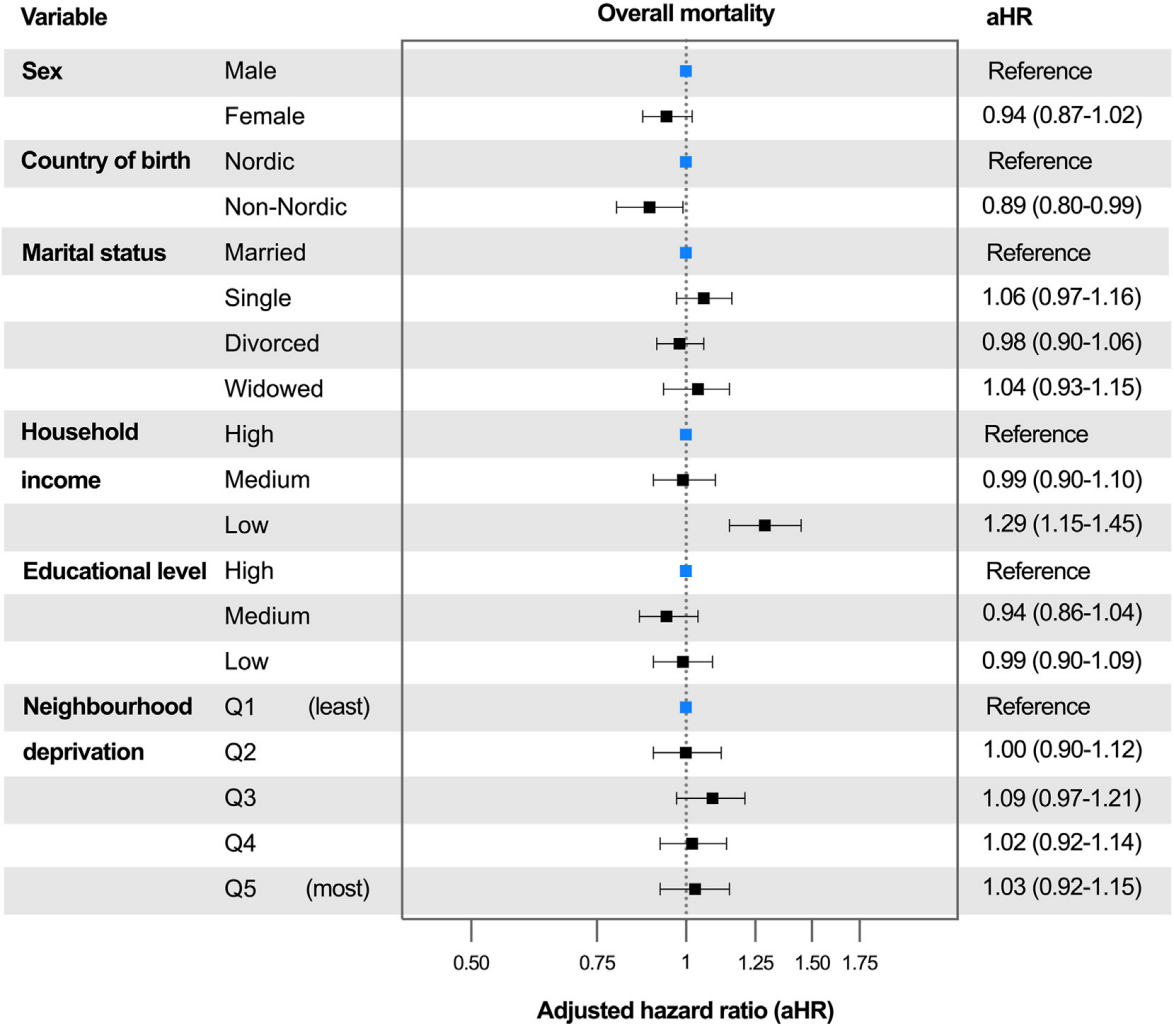
Results from multivariable Cox regression model showing adjusted hazard ratios for overall mortality in patients with hepatocellular carcinoma in Sweden from 2011 to 2021. The multivariable model was adjusted for all variables shown, as well as age, year of diagnosis, aetiology, cirrhosis status (no cirrhosis, compensated, decompensate d), tumour size, number, tumour thrombosis, metastasis, performance status, and comorbidities (arterial hypertension, type 2 diabetes, coronary artery disease, cerebrovascular disease, chronic kidney disease, chronic obstructive pulmonary disease).

**Fig. 3:**
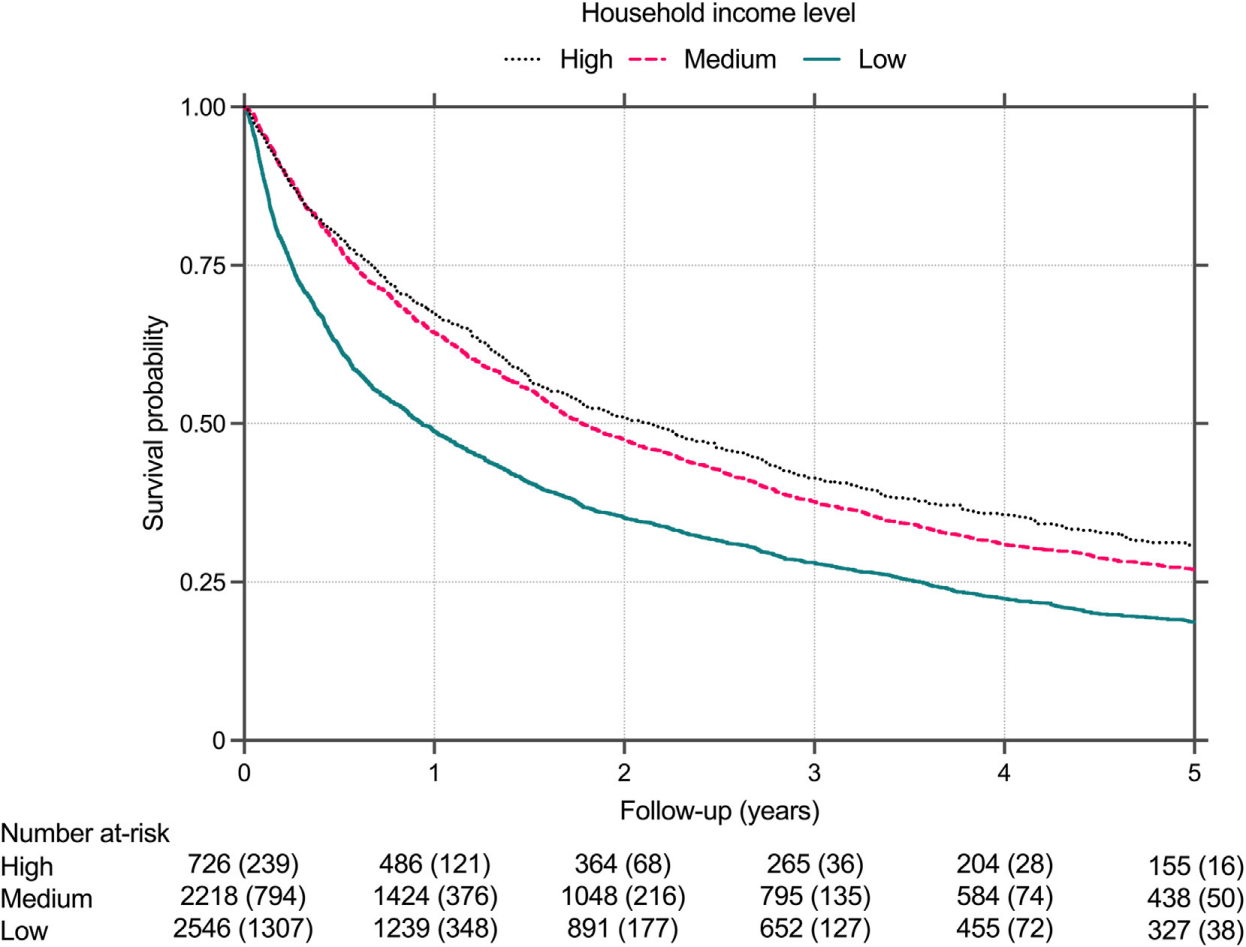
Survival curves of patients with low, medium, and high household income diagnosed with hepatocellular carcinoma in Sweden between 2011 and 2021. Results from a multivariable Cox regression model adjusted for sex, age at diagnosis, country of birth, marital status, educational level, neighbourhood deprivation level, year of diagnosis, aetiology, cirrhosis status (no cirrhosis, compensated, decompensated), tumour size, number, tumour thrombosis, metastasis, performance status, and comorbidities (arterial hypertension, type 2 diabetes, coronary artery disease, cerebrovascular disease, chronic kidney disease, chronic obstructive pulmonary disease). The number of terminal events is shown in parentheses. Time after HCC diagnosis was limited to 5 years.

After full adjustment, low household income was associated with an increased mortality risk (aHR 1·29; 95% CI: 1·15–1·45) compared to high household income (Figs. 2 and 3), and with an aHR of 1·28 (95% CI: 1·20–1·36) compared to medium household income. Unadjusted and adjusted estimates for all included variables are presented in Table S11.

### Trend analysis: aetiologies and HCC outcomes

**Fig. 4:**
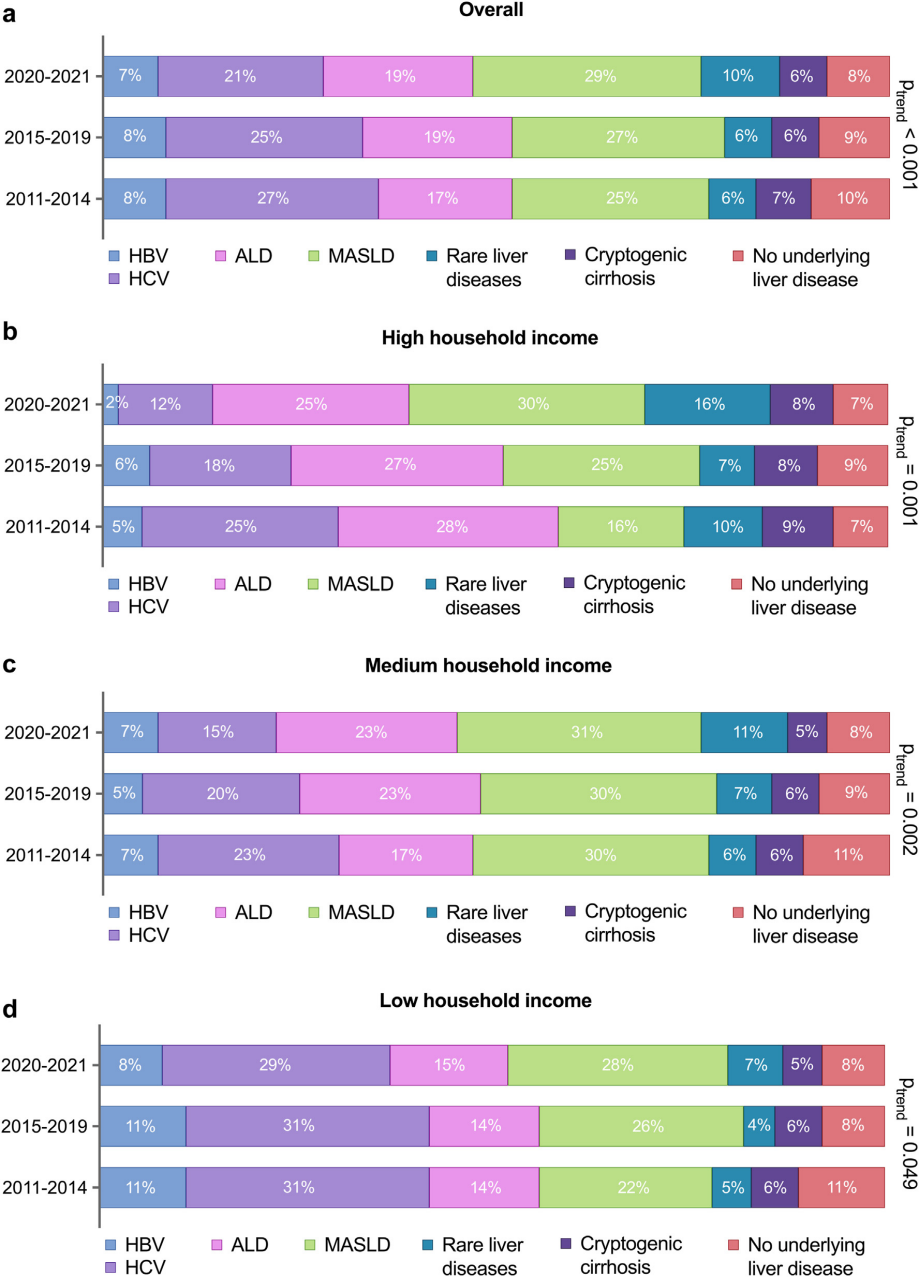
Underlying aetiologies in patients diagnosed with hepatocellular carcinoma in Sweden across three time periods. Trend analyses with corresponding p-values are presented for the overall cohort (a) and by household income groups (b–d). ALD: alcohol-related liver disease; HBV: hepatitis B virus; HCV: hepatitis C virus; MASLD: metabolic dysfunction-associated steatotic liver disease.

The sociodemographic characteristics and the proportion of patients with underlying cirrhosis remained consistent across all time periods. However, aetiology distribution shifted, with MASLD becoming the leading cause of HCC in 2014. The prevalence of viral hepatitis declined markedly in medium- and high-income groups but only slightly in the low-income group (Fig. 4).

Compared to patients diagnosed in 2011–2014, those diagnosed with HCC in 2015–2019 and 2020–2021 had a higher likelihood of diagnosis via surveillance (aOR 1.84, 95% CI: 1.56–2.16; and aOR 1.96, 95% CI: 1.60–2.41) and early-stage HCC diagnosis (aOR 1.39, 95% CI: 1.21–1.60; and aOR 1.43, 95% CI: 1.20–1.70). The likelihood of receiving curative treatment did not differ significantly between periods, but mortality risks were lower for patients diagnosed in 2015–2019 (aHR 0.90, 95% CI: 0.84–0.96) and 2020–2021 (aHR 0.89, 95% CI: 0.81–0.98) compared to 2011–2014. No statistically significant differences in HCC outcomes were found between patients diagnosed in 2015–2019 and 2020–2021.

### Sensitivity analyses

The test of proportionality yielded a p-value of 0.097 for the global Cox model. Log–log survival plots for household income level (Figure S2) suggested that survival differences were more pronounced between 0.5 and 2 years after diagnosis. To further investigate potential time-varying effects, we modelled household income as a time-dependent variable. This analysis showed that the gradient in survival differences increased slightly with follow-up time, but the results (Table S11) remained largely consistent with the primary model. Given the minimal differences observed, we retained the original model for clarity and interpretability.

For patients with ECOG 0–1, low household income was associated with lower odds of diagnosis via surveillance (aOR 0.74, 95% CI: 0.59–0.93 vs. high income; aOR 0.76, 95% CI: 0.61–0.93 vs. medium income), early-stage HCC diagnosis (aOR 0.75, 95% CI: 0.58–0.95; aOR 0.75, 95% CI: 0.60–0.89), and receipt of curative treatment (aOR 0.58, 95% CI: 0.42–0.81; aOR 0.73, 95% CI: 0.59–0.91). Low income was also linked to higher mortality (aHR 1.40, 95% CI: 1.20–1.62; aHR 1.32, 95% CI: 1.19–1.47).

For patients with ECOG ≥2, low income was associated with lower odds of diagnosis via surveillance (aOR 0.50, 95% CI: 0.35–0.90; aOR 0.77, 95% CI: 0.55–1.09) and early-stage HCC diagnosis (aOR 0.41, 95% CI: 0.25–0.68; aOR 0.51, 95% CI: 0.37–0.71), but not with curative treatment or mortality risk.

## Discussion

This nationwide study highlights the substantial impact of SES on early diagnosis, treatment, and survival outcomes in patients with HCC in Sweden. Our findings show that individual-level SES, assessed by household income, strongly influences HCC outcomes from several perspectives. Lower income was consistently associated with a reduced likelihood of HCC diagnosis through surveillance, lower odds of early-stage diagnosis, reduced chances of receiving curative treatment, and poorer survival. No other SES indicator exanimated here was associated with HCC outcomes after adjusting for household income, suggesting that household income is the primary SES determinant of health inequities in Sweden.

Our results mirror trends observed in other European regions, such as in the UK and Italy, where SES strongly influences HCC risk factors, diagnostic delays, and treatment pathways.^23^^,^^24^ Similarly, non-European countries, including the US, report comparable SES-related health disparities in HCC.^25^ However, unlike the US, Sweden benefits from a healthcare infrastructure that provides near-universal access to care to a low cost for patients. Still, our findings indicate that socioeconomic disparities persist due to barriers beyond financial access. Patients from low socioeconomic backgrounds may face challenges in navigating the healthcare system, lower health literacy, higher comorbidity burden, and implicit biases in clinical decision-making. Additionally, differences in healthcare seeking behaviour and physician-patient communication may contribute to variations in treatment access.

Despite favourable trends in surveillance, early-stage diagnosis and mortality outcomes over time, only a quarter of patients are diagnosed with HCC via surveillance, the likelihood of receiving curative treatment has not improved, and health inequities persist. Individuals with low household income not only have the highest incidence of HCC in Sweden,^12^ but are also more likely to underdiagnosed liver cirrhosis,^11^ underscoring the need for improved HCC surveillance in this group.^26^ Additionally, patients with low income have higher ECOG at diagnosis, which directly affects treatment decisions and survival. Low household income remained consistently associated with worse outcomes across all aspect examined, even after adjustment for ECOG. Sensitivity analyses further supported this, showing that low household income was associated with worse outcomes in patients with ECOG 0–1, but not in those with ECOG ≥2, likely reflecting the reduced relevance of SES in patients with a poor prognosis from the outset.

We have identified significant differences in HCC aetiology and trends by household income. The declining proportion of HCV-related HCC in our study aligns with the widespread implementation of direct-acting antiviral treatment.^27^ However, while MASLD now is the main cause of HCC in patients with medium and high household income, the prevalence of underlying viral hepatitis among patients with low household income has scarcely declined. This underscores the needed for increased efforts to improve access to these treatments for the most socioeconomically deprived groups.^8^^,^^28^

Variations in HCC aetiology across socioeconomic groups may help explain some of the disparities observed in diagnosis, HCC stage and treatment. Patients with viral hepatitis or ALD may be less prone to seek medical care. Further, viral hepatitis, especially HBV, is more prevalent in immigrants. Also, the overall incidence of HCC in cirrhosis varies by underlying liver disease aetiology, being estimated at 23 per 1000 person-years in Sweden, with 5- and 10-year cumulative incidences of 8% and 12%, respectively.^29^ Viral hepatitis-related cirrhosis was found to have highest incidence of HCC (41 per 1000 person-years), and men with viral hepatitis had the highest 5- and 10-year cumulative incidences of HCC: 18% and 27%, respectively.

While the variation in aetiologies observed between different SES groups is striking, adjustments for aetiology in multivariate analyses did not significantly change aORs and aHRs estimates, indicating that aetiology may only partially explain outcome differences observed between SES groups. Other important clinical factors such as inclusion into surveillance programs, liver disease severity at diagnosis, and comorbidity may play similarly significant roles.

Healthcare providers, especially hepatologists treating patients from low SES backgrounds, should be made aware of the higher risk this group faces for poorer HCC outcomes. Training and awareness programs could help physicians better identify and prioritize interventions for these vulnerable patients, particularly in surveillance, early detection, and treatment.

In summary, our findings emphasize the detrimental impact of low household income on multiple HCC outcomes. Low household income, rather than country of birth, is the most significant factor influencing HCC outcomes. Efforts to address health inequities should focus on low-income areas rather than specific immigrant populations. The most deprived neighbourhoods, with high proportions of residents in low-income households, may be key areas for targeted HCC and liver disease awareness campaigns aimed at both healthcare providers and the general public.

Future research is crucial to understanding the underlying mechanisms contributing to poorer outcomes in low-income patients. Investigating these factors will offer valuable insights into why low-income individuals experience significantly worse outcomes and inform targeted interventions to improve care for this high-risk group. By promoting timely HCC diagnosis and treatment in disadvantaged populations, these efforts could significantly improve survival outcomes and reduce the overall burden of HCC.

Our analyses are based on data from over 95% of all HCC cases in Sweden over a long but modern study period.^13^ The risk of selection bias is low due to universal access to healthcare services in the country. We used additional cross-linked national registries to provide additional information on important parameters.^19^^,^^30^ The integration of detailed data from SweLiv with these registries enhances the comprehensiveness of our findings compared to typical register-based studies. Additional strengths include: (a) no missing data related on critical variables, including country of birth, treatments, and HCC staging; (b) use of well-defined variables and robust statistical methods, producing consistent findings; and (c) inclusion of country of birth and marital status alongside individual- and contextual-level SES indicators, providing a nuanced understanding of sociodemographic influences.

Some limitations should be noted. First, our classification of country of birth does not allow for comparisons among different nationalities or ethnicities. Second, data from SweLiv on surveillance parameters including surveillance interval and quality was limited. Third, since this study did not aim to evaluate the impact of surveillance on survival, potential length and lead-time biases should be considered when interpreting the survival rate estimates. Still, prior research, including a large meta-analysis,^26^ has consistently demonstrated that HCC diagnosed through surveillance is associated with earlier-stage detection, increased receipt of curative treatment, and improved survival. Similar findings have been reported in our own previous research.^11^ Given this extensive prior evidence, our study did not focus on confirming the benefits of surveillance.

In our cohort, 24% of patients were classified as non-cirrhotic, which aligns with previous findings from Swedish registry studies.^11^ The classification of cirrhosis was based on data from SweLiv and the National Patient Register, which we combined to improve accuracy. However, as with any registry-based classification, some cases may have been misclassified due to undocumented cirrhosis or limitations in clinical reporting.

To account for potential differences between cirrhotic and non-cirrhotic patients, we adjusted for cirrhosis status in our multivariable models. This adjustment did not significantly alter the estimated aORs or aHRs for SES-related disparities, suggesting that socioeconomic differences in surveillance inclusion, disease severity at diagnosis, treatment access, and survival are independent of cirrhosis status. Given these findings, we did not perform a detailed subgroup analysis comparing cirrhotic and non-cirrhotic patients, as it was beyond the scope of this study, which primarily focuses on socioeconomic disparities in HCC care and outcomes.

This study highlights critical disparities in HCC care across SES groups, driven primarily by low household income level, which adversely affects all stages of HCC management. These findings underscore the urgent need for targeted public health strategies that address the multifaceted barriers faced by socioeconomically disadvantaged populations, ultimately aiming to bridge the gap in HCC management and improve health equities.

## Contributors

The work reported in the article has been performed by the authors, unless clearly specified in the text. Specific author contributions: JV: Conceptualization, Methodology, Formal analysis, Investigation, Data acquisition and curation, Visualization, Writing—Original Draft. HH: Conceptualization, Methodology, Investigation, Writing-Original Draft, Supervision. MSE and MR: Conceptualization, Writing-review and editing. US: Conceptualization, Methodology, Data acquisition, Writing—Original Draft, Funding acquisition, Supervision. JV and US have direct access to the data. All authors have contributed and approved the final version.

## Data sharing statement

Datasets generated and analysed during the current study are not publicly available due to legal restrictions, but additional analyses may be requested to the corresponding author upon reasonable request.

## Declaration of interests

JV has received consulting fees from Roche and Astra Zeneca and a research grant from Eisai. HH:s institutions have received research funding from Astra Zeneca, EchoSens, Gilead, Intercept, MSD, Novo Nordisk and Pfizer. HH has served as consultant or on advisory boards for Astra Zeneca, Bristol Myers-Squibb, MSD and Novo Nordisk and HH has been part of hepatic events adjudication committees for Ar, Boehringer Ingelheim, KOWA and GW Pharma. MSE has received consulting fees from Baxter, Astra Zeneca and Eisai. MR is the Chair of the National Guideline Group for Hepatocellular Carcinoma. US has no conflicts of interest.
