# Supplementary material for: Socioeconomic inequalities in diagnostics, care and survival outcomes for hepatocellular carcinoma in Sweden: a nationwide cohort study
Source: Lancet Reg Health Eur. 2025 Mar 20;52:101273. doi: 10.1016/j.lanepe.2025.101273 (PMC11987686; doi:10.1016/j.lanepe.2025.101273)
Supplement: Supplementary Figures and Tables [file mmc1.pdf]

Socioeconomic inequalities in diagnostics, care and survival outcomes for hepatocellular carcinoma in Sweden: a nationwide cohort study

Juan Vaz, Hannes Hagström, Malin Sternby Eilard, Magnus Rizell, Ulf Strömberg

Supplementary data

Table of contents

Table S1	Definitions …………………………………………………………………..	2
Table S2	Baseline characteristics by sex ……………………………………….………...	5
Table S3	Baseline characteristics by country of birth ………………………………………	7
Table S4	Baseline characteristics by marital status ………….…………………………..	9
Table S5	Baseline characteristics by educational level ……………………………………..	11
Table S6	Baseline characteristics by neighbourhood deprivation level …………………….	13
Table S7	Baseline characteristics by cirrhosis status ……………………….……………..	15
Table S8	Likelihood of HCC diagnosis during surveillance ………………………………..	17
Table S9	Likelihood of HCC diagnosis at early-stage ……………………………………	18
Table S10	Likelihood of receiving curative treatment after HCC diagnosis …………………	19
Table S11	Mortality risk after HCC diagnosis ……………………………………………….	20
Figure S1	Swedish treatment algorithm for hepatocellular carcinoma ………………………	21
Figure S2	Log-log plot of survival …………………………………………………………..	22

Table S1. List of variables and definitions

**Sociodemographic characteristics**	
Sex	Male or female according to the Swedish population register.
Age at diagnosis	Age in years.
Country of birth	Nordic: born in Sweden, Denmark, Finland, Norway, or Iceland. Non-Nordic: born outside Nordic countries (Source: Statistics Sweden)
Marital status	Married, single, divorced/separated, or widowed
Household income	Based on the distribution of household incomes for all households in Sweden. Low (lowest quartile, <25%), medium (second and third quartiles, 25-75%), high (highest quartile, >75%). Selected for the year prior to cancer diagnosis
Educational level	Based on years of formal education. Low (≤9 years), medium (10-12 years), high (>12 years)
Neighbourhood deprivation	Based on the distribution of individuals with a low household income (as defined above) living Demographic Statistical Areas (DeSOs; n = 5984) in Sweden. Proportions of inhabitants with low household income were calculated for each DeSO, which were thereby sorted for least deprived DeSOs to most deprived. Based on this ranking we allocated each DeSO into national quintiles (Q1-Q5), each quintile comprising approximately 1197 different DeSOs, from least deprived (Q1) to most deprived (Q5).
**Aetiologies**	
Hepatitis B	ICD-10: B16, B17.0, B18.0, B18.1 *OR* specified as the main cause of HCC in SweLiv
Hepatitis C	ICD-10: B17.1, B18.2 *OR* specified as the main cause of HCC in SweLiv
ALD	ICD-10: K70 *OR* alcohol use disorder *AND* no other known liver disease, ICD-10: E24.4, F10, G31.2, G62.1, G72.1, I42.6, K29.2, K85.2, K86.0, R78.0, T51.0, T51.9, Y57.3, Y90, X65, Z71.4, Z72.1 *OR* ATC: N07BB01 (disulfiram), N07BB03 (acamprosat), N07BB04 (naltrexon) *OR* specified as the main cause of HCC in SweLiv
MASLD	ICD-10: K75.8, K76.0 *OR* obesity (ICD-10: E66) *AND/OR* diabetes (as defined below) *OR* specified as the main cause of HCC in SweLiv
Other liver diseases	ICD-10: K75.4, K83.0A, K74.3, E83.0B, E88.0A, E88.0B, E83.1, K73.9, K73.2, I82.0, K76.5, K74.4, K74.5 *OR* specified as the main cause of HCC in SweLiv
Cryptogenic cirrhosis	ICD-10: K74.6 *AND* no diagnosed liver disease *OR* specified as the main cause of HCC in SweLiv
**Liver cirrhosis**	ICD-10: B18.0E, B18.0G, B18.1E, B18.1G, B18.2E, B18.2G, B18.8E, B18.8G, B18.9E, B18.9G, G93.4, I85.0 I85.9, I98.2, I98.3, K70.3, K71.7, K74.6, K76.6, K76.7, R18. (no heart failure [I50, I11.0, I13.0, I13.2] or cancer other than HCC). Procedural: JCA20, JCA22, JCA32, JDA22 *OR* TJA10, TJA40 *AND* known liver disease *OR* specified as having cirrhosis in SweLiv
Compensated	Child-Pugh ≤ 7
Decompensated	Child-Pugh >7 OR ascites (ICD-10: R18) OR oesophageal bleeding (ICD-10: I85.0, I98.3) OR hepatorenal syndrome (ICD-10: K76.7)
Child-Pugh score	Bilirubin (µmol/L): <34.2 (+1), 34.2-51.3 (+2), >51.3 (+3)<br>Albumin (g/L): >35 (+1), 28-35 (+2), <28 (+3)<br>Prothrombin time (international normalized ratio): <1.7 (+1), 1.7-2.2 (+2), >2.2 (+3); not used for patients treated with warfarin<br>Ascites: absent (+1), slight (+2), moderate (+3)<br>Encephalopathy: none (+1), grade 1-2 (+2), grade 3-4 (+3)<br><br>Calculation for missing data:<br>1 laboratory parameter missing: CP ≤ 7 if the sum of other parameters = 4<br>2-3 laboratory parameters missing: CP > 7 if the sum of other parameters ≥ 6<br>Encephalopathy grade missing: +1 if the sum of other parameters = 4 and ECOG =0<br>Ascites grade missing: +1 if the sum of other parameters =4 and ECOG =0
**Diagnostic pathway**	Surveillance: patient with known liver disease included in the surveillance program for HCC<br>Clinical symptoms: diagnosed with HCC during a clinical work-up related to liver disease-associated symptoms

	Incidental: HCC diagnosis on passant in surgery or radiology in a patient without clinical symptoms related to liver disease<br><br>Diagnostic pathway for missing data:<br>Clinical symptoms: if no prior liver disease was registered and ICD-10 codes such as R18 (ascites) or R10.0 (abdominal pain), among others, were registered at the time of HCC diagnosis<br>Incidental: if no prior liver disease was registered and no other symptom-related ICD-10 codes were registered at the time of HCC diagnosis
**ECOG performance status**	0: Fully active, able to carry on all pre-disease performance without restriction.<br>1: Restricted in physically strenuous activity but ambulatory and able to perform work of a light or sedentary nature.<br>2: Ambulatory and capable of all self-care but unable to carry out any work activities; up and about >50% of waking hours.<br>3: Capable of only limited self-care; confined to bed or chair >50% of waking hours.<br>4: Completely disabled; cannot conduct any self-care; totally confined to bed or chair.
**Tumour size**	Largest diameter in millimetres: ≤ 20, 21-30, >30
**Tumour number**	Single, 2-3, >3 (multinodular)
**Lymph node metastasis**	TNM classification for HCC 8th Edition: N0, N1, NX
**Extrahepatic metastasis**	TNM classification for HCC 8th Edition: M0, M1, MX
**Tumour thrombosis**	Yes/No
**HCC Stage**	Early:<br>1) Single tumour or up to 3 nodules ≤3 cm *AND*<br>2) N0, M0 *AND*<br>3) ECOG PS 0-1 (2 in transplant/resection candidates) *AND*<br>4) Child-Pugh score ≤7 (not necessary if transplant candidate)<br><br>Intermediate:<br>1) Multinodular tumours *AND*<br>2) N0, M0 *AND*<br>3) ECOG PS 0-1 (2 in transplant/resection candidates) *AND*<br>4) Child-Pugh score ≤7 (not necessary if transplant candidate)<br><br>Advanced:<br>1) M1 *OR* N1 *AND*<br>2) ECOG PS 0-1 (2 in transplant/resection candidates) *AND*<br>3) Child-Pugh score ≤7 (not necessary if transplant candidate)<br><br>Terminal:<br>1) Child-Pugh score >7 *OR*<br>2) ECOG ≥ 2 *AND*<br>3) No candidate for liver transplantation *AND*<br>4) Severe comorbidity burden
**Comorbidities**	
Arterial hypertension	ICD-10: I10-I15

Diabetes	ICD-10: E10-E14; ATC: A10A (insulin), A10B (other glucose lowering drugs), A10X (other drugs against diabetes)
Coronary artery disease	ICD-10: I20-I25; ATC: C01DA (organic nitrates)
Cerebrovascular disease	ICD-10: I60-I69
Chronic kidney disease	ICD-10: N11, N18, I13.1, I13.2, I13.9, I12.0, Z94.0, T86.1, Z49, Z99.2
COPD	ICD-10: J41-J44 (40 years and older)
**Treatment**	
Curative	Transplantation: if registered at any point, regardless of previous treatments. ICD-10 Z94.4, T86.4; Procedural: JCA20, JCA22, JCA32, JDA22, JJC
	Resection: if performed before ablation. Procedural: JJB, JJA40, JJA41
	Ablation: if performed before resection. Procedural: JJA43, JJA44, TJJ10,
Palliative	Transarterial chemoembolisation
	Systemic chemo- or immunotherapy registered in SweLiv *AND/OR* ATC: L01EX02 (sorafenib), L01EX08 (lenvatinib) *AND/OR* ATC L01EX05 (regorafenib)
Best supportive care	No antitumour treatment reported

ALD: alcohol-related liver disease; ATC: Anatomical Therapeutic Chemical Classification System; COPD: Chronic obstructive pulmonary disease; ECOG PS = Eastern Cooperative Oncology Group performance status; HCC = Hepatocellular carcinoma; Household income = Disposable income per household per consumption unit; ICD-10: International Classification of Diseases; MASLD: Metabolic dysfunction-associated steatotic liver disease; M: Extrahepatic metastasis; N: Regional lymph node metastasis. SweLiv: Swedish quality register for cancers found in the liver, gallbladder and bile ducts.

Household income refers to a person's disposable income per consumption unit. Disposable income is the sum of all taxable and tax-free income minus taxes and negative transfers. The income includes gains/losses, i.e. the gain/loss arising from a sale (realization) of assets, for example, stocks, mutual funds or real estate. To compare disposable income and economical purchasing power between different household types, a weight system is used where consumption is related to household composition. Disposable income is divided by the weight of consumption of the household. The scale is determined by Statistics Sweden and is based on, among other things, budget calculations carried out by the Swedish Consumer Agency and the basis for assessing a basic consumption that can be calculated for different household types. The consumption unit scale used by Statistics Sweden is the following: single or living alone 1.0; cohabiting couple 1.51; additional adult 0.6; first child 0-19 years 0.52; second and subsequent children 0-19 years 0.42.

Table S2. Baseline characteristics of 5,490 patients diagnosed with hepatocellular carcinoma in Sweden between 2011 and 2021, stratified by sex

	Sex		
	**Male**	**Female**	**Total**
	4179 (76)	1311 (24)	5490 (100)
**Median age**	69 (62-76)	72 (64-78)	70 (62-76)
**Country of birth**			
Nordic	3571 (85)	1123 (86)	4694 (85)
Non-Nordic	608 (15)	188 (14)	796 (15)
**Marital status**			
Married	2009 (48)	504 (38)	2513 (46)
Single	923 (22)	173 (13)	1096 (20)
Divorced/separated	928 (22)	299 (23)	1227 (22)
Widowed	319 (8)	335 (26)	654 (12)
**Household income**			
High	572 (14)	154 (12)	726 (13)
Medium	1736 (41)	482 (37)	2218 (41)
Low	1871 (45)	675 (51)	2546 (46)
**Educational level**			
High	671 (16)	224 (17)	895 (16)
Medium	1860 (44)	531 (41)	2391 (44)
Low	1565 (38)	515 (39)	2080 (38)
Unknown	83 (2)	41 (3)	124 (2)
**Neighbourhood deprivation**			
Q1 (least)	547 (13)	155 (12)	702 (13)
Q2	722 (17)	222 (17)	944 (17)
Q3	814 (20)	279 (21)	1093 (20)
Q4	976 (23)	264 (20)	1240 (23)
Q5 (most)	1120 (27)	391 (30)	1498 (27)
**Aetiology**			
Hepatitis B	357 (9)	83 (6)	440 (8)
Hepatitis C	1113 (27)	247 (19)	1360 (25)
Alcohol-related liver disease	900 (21)	130 (10)	1030 (19)
MASLD	1103 (26)	364 (28)	1467 (27)
Other liver diseases	179 (4)	184 (14)	363 (6)
Cryptogenic cirrhosis	238 (6)	103 (8)	341 (6)
No diagnosed liver disease	289 (7)	200 (15)	489 (9)
**Liver cirrhosis**	3251 (78)	913 (70)	4164 (76)
Child-Pugh ≤ 7[a]	2487 (75)	698 (76)	3185 (76)
Child-Pugh >7[a]	764 (25)	215 (24)	979 (24)
**Diagnostic pathway**			
Surveillance	1063 (26)	338 (26)	1401 (26)
Clinical symptoms	2191 (52)	689 (53)	2880 (52)
Incidental finding	761 (18)	228 (17)	981 (18)
Missing	164 (4)	56 (4)	220 (4)
**ECOG performance status**			
0	1308 (31)	361 (28)	1669 (30)
1	1270 (30)	421 (32)	1691 (31)
≥2	1601 (39)	529 (40)	2130 (39)
**Tumour size (mm)**			
Median	45 (25-80)	44 (25-80)	45 (25-80)
≤20	700 (17)	242 (18)	942 (17)
21-29	619 (15)	203 (16)	822 (15)
≥30	2860 (68)	866 (66)	3726 (68)
**Number of tumours**			
1	2171 (52)	806 (61)	2977 (54)
2-3	981 (23)	270 (21)	1251 (23)
>3	916 (22)	197 (15)	1113 (20)
Uncertain	111 (3)	38 (3)	149 (3)
**Lymph node metastasis**	471 (11)	139 (11)	610 (11)
**Extrahepatic metastasis**	615 (15)	184 (14)	799 (15)
**Tumour thrombosis**	544 (13)	164 (13)	708 (13)
**Stage at diagnosis**			
Early	1412 (34)	493 (38)	1905 (35)
Intermediate	860 (21)	278 (21)	1138 (21)
Advanced	550 (13)	141 (11)	691 (12)
Terminal	1357 (32)	399 (30)	1756 (32)
**Comorbidities**			
Arterial hypertension	1542 (37)	493 (38)	2035 (37)
Type 2 diabetes	1859 (44)	495 (38)	2354 (43)
Coronary artery disease	819 (20)	189 (14)	1008 (18)
Cerebrovascular disease	476 (11)	116 (9)	592 (11)
Chronic kidney disease	356 (9)	89 (7)	443 (8)
COPD	551 (13)	172 (13)	723 (13)
**Treatment**			

Transplantation	281 (7)	69 (5)	350 (6)
Resection	618 (15)	238 (18)	856 (15)
Ablation	641 (15)	218 (17)	859 (16)
Palliative	1204 (29)	367 (28)	1571 (29)
Best supportive care	1435 (34)	419 (32)	1854 (34)

COPD: chronic obstructive pulmonary disease; ECOG PS: Eastern Cooperative Oncology Group; MASLD: metabolic dysfunction-associated steatotic liver disease. [a] Percentage of patients with cirrhosis.

Table S3. Baseline characteristics of 5,490 patients diagnosed with hepatocellular carcinoma in Sweden between 2011 and 2021, stratified by country of birth

	Country of birth		
	Nordic	Non-Nordic	Total
	4694 (85)	796 (15)	5490 (100)
**Male sex**	3571 (76)	608 (76)	4179 (76)
**Median age**	71 (63-77)	65 (56-73)	70 (62-76)
**Marital status**			
Married	2075 (44)	438 (55)	2513 (46)
Single	1017 (22)	79 (10)	1096 (20)
Divorced/separated	1021 (22)	206 (26)	1227 (22)
Widowed	581 (12)	73 (9)	654 (12)
**Household income**			
High	664 (14)	62 (8)	726 (13)
Medium	1953 (42)	265 (33)	2218 (41)
Low	2077 (44)	469 (59)	2546 (46)
**Educational level**			
High	727 (15)	168 (21)	895 (16)
Medium	2113 (45)	278 (35)	2391 (44)
Low	1820 (39)	260 (33)	2080 (38)
Unknown	34 (1)	90 (11)	124 (2)
**Neighbourhood deprivation**			
Q1 (least)	640 (14)	62 (8)	702 (13)
Q2	871 (18)	73 (9)	944 (17)
Q3	983 (21)	110 (14)	1093 (20)
Q4	1087 (23)	153 (19)	1240 (23)
Q5 (most)	1113 (24)	398 (50)	1498 (27)
**Aetiology**			
Hepatitis B	193 (4)	247 (31)	440 (8)
Hepatitis C	1152 (25)	208 (26)	1360 (25)
Alcohol-related liver disease	950 (20)	80 (10)	1030 (19)
MASLD	1311 (28)	156 (20)	1467 (27)
Other liver diseases	335 (7)	28 (3)	363 (6)
Cryptogenic cirrhosis	304 (6)	37 (5)	341 (6)
No diagnosed liver disease	449 (10)	40 (5)	489 (9)
**Liver cirrhosis**	3493 (74)	671 (84)	4164 (76)
Child-Pugh ≤ 7[a]	2684 (77)	501 (75)	3185 (76)
Child-Pugh >7[a]	809 (23)	170 (25)	979 (24)
**Diagnostic pathway**			
Surveillance	1151 (25)	250 (31)	1401 (26)
Clinical symptoms	2487 (53)	393 (49)	2880 (52)
Incidental finding	865 (18)	124 (16)	981 (18)
Missing	191 (4)	29 (4)	220 (4)
**ECOG performance status**			
0	1362 (29)	307 (39)	1669 (30)
1	1444 (31)	247 (31)	1691 (31)
≥2	1888 (40)	242 (30)	2130 (39)
**Tumour size (mm)**			
Median	45 (25-80)	40 (23-70)	45 (25-80)
≤20	777 (16)	165 (21)	942 (17)
21-29	689 (15)	136 (17)	822 (15)
≥30	3231 (69)	495 (62)	3726 (68)
**Number of tumours**			
1	2547 (54)	430 (54)	2977 (54)
2-3	1067 (23)	184 (23)	1251 (23)
>3	947 (20)	166 (21)	1113 (20)
Uncertain	133 (3)	16 (2)	149 (3)
**Lymph node metastasis**	540 (11)	70 (9)	610 (11)
**Extrahepatic metastasis**	699 (15)	100 (12)	799 (15)
**Tumour thrombosis**	601 (13)	107 (13)	708 (13)
**Stage at diagnosis**			
Early	1582 (34)	323 (41)	1905 (35)
Intermediate	960 (20)	178 (22)	1138 (21)
Advanced	574 (12)	117 (15)	691 (12)
Terminal	1578 (34)	178 (22)	1756 (32)
**Comorbidities**			
Arterial hypertension	1803 (38)	232 (29)	2035 (37)
Type 2 diabetes	2034 (43)	320 (40)	2354 (43)
Coronary artery disease	903 (19)	105 (13)	1008 (18)
Cerebrovascular disease	535 (11)	57 (7)	592 (11)
Chronic kidney disease	390 (8)	55 (7)	443 (8)
COPD	660 (14)	63 (8)	723 (13)
**Treatment**			

Transplantation	251 (5)	99 (12)	350 (6)
Resection	730 (16)	126 (16)	856 (15)
Ablation	734 (16)	215 (16)	859 (16)
Palliative	1319 (28)	252 (32)	1571 (29)
Best supportive care	1660 (35)	194 (24)	1854 (34)

COPD: chronic obstructive pulmonary disease; ECOG: Eastern Cooperative Oncology Group; MASLD: metabolic dysfunction-associated steatotic liver disease. [a] Percentage of patients with cirrhosis.

Table S4. Baseline characteristics of 5,490 patients diagnosed with hepatocellular carcinoma in Sweden between 2011 and 2021, stratified by marital status

	Marital status				
	**Married**	**Single**	**Divorced**	**Widowed**	**Total**
	2513 (46)	1096 (20)	1227 (22)	654 (12)	5490 (100)
**Male sex**	2009 (80)	923 (84)	928 (76)	319 (49)	4179 (76)
**Median age**	71 (65-77)	63 (57-70)	67 (61-73)	79 (74-83)	70 (62-76)
**Country of birth**					
Nordic	2075 (83)	1017 (93)	1021 (83)	581 (89)	2513 (46)
Non-Nordic	438 (17)	79 (7)	206 (17)	73 (11)	1096 (20)
**Household income**					
High	493 (20)	83 (8)	101 (8)	49 (7)	726 (13)
Medium	1215 (48)	344 (31)	438 (36)	221 (34)	2218 (41)
Low	805 (32)	669 (61)	688 (56)	384 (59)	2546 (46)
**Educational level**					
High	511 (20)	111 (10)	190 (15)	83 (13)	895 (16)
Medium	1014 (40)	548 (50)	585 (48)	244 (37)	2391 (44)
Low	920 (37)	417 (38)	438 (36)	305 (47)	2080 (38)
Unknown	68 (3)	20 (2)	14 (1)	22 (3)	124 (2)
**Neighbourhood deprivation**					
Q1 (least)	421 (17)	97 (9)	111 (9)	73 (11)	702 (13)
Q2	499 (20)	170 (15)	181 (15)	94 (14)	944 (17)
Q3	524 (21)	183 (17)	232 (19)	154 (24)	1093 (20)
Q4	514 (20)	286 (26)	286 (23)	154 (24)	1240 (23)
Q5 (most)	555 (22)	360 (33)	417 (34)	179 (27)	1498 (27)
**Aetiology**					
Hepatitis B	195 (8)	110 (10)	115 (9)	20 (3)	440 (8)
Hepatitis C	394 (16)	467 (43)	444 (36)	55 (9)	1360 (25)
Alcohol-related liver disease	509 (20)	180 (16)	256 (21)	85 (13)	1030 (19)
MASLD	791 (31)	186 (17)	213 (17)	277 (42)	1467 (27)
Other liver diseases	195 (8)	44 (4)	69 (6)	55 (9)	363 (6)
Cryptogenic cirrhosis	197 (8)	43 (4)	47 (4)	54 (8)	341 (6)
No diagnosed liver disease	232 (9)	66 (6)	83 (7)	108 (16)	489 (9)
**Liver cirrhosis**	1843 (73)	914 (83)	1005 (82)	402 (61)	4164 (76)
Child-Pugh ≤ 7[a]	1418 (77)	687 (75)	772 (77)	308 (77)	3185 (76)
Child-Pugh >7[a]	425 (23)	225 (25)	233 (23)	94 (23)	979 (24)
**Diagnostic pathway**					
Surveillance	613 (24)	322 (29)	375 (31)	91 (14)	1401 (26)
Clinical symptoms	1343 (54)	558 (51)	603 (49)	376 (58)	2880 (52)
Incidental finding	454 (18)	170 (16)	207 (17)	158 (24)	981 (18)
Missing	103 (4)	46 (4)	42 (3)	29 (4)	220 (4)
**ECOG performance status**					
0	832 (33)	361 (33)	360 (29)	116 (18)	1669 (30)
1	775 (31)	312 (28)	399 (33)	205 (31)	1691 (31)
≥2	906 (36)	423 (39)	468 (38)	333 (51)	2130 (39)
**Tumour size (mm)**					
Median	48 (25-85)	40 (23-75)	40 (25-75)	50 (30-100)	45 (25-80)
≤20	413 (16)	214 (20)	231 (19)	84 (13)	942 (17)
21-29	352 (14)	181 (16)	208 (17)	81 (12)	822 (15)
≥30	1748 (70)	701 (64)	788 (64)	489 (75)	3726 (68)
**Number of tumours**					
1	1388 (55)	544 (50)	639 (52)	406 (62)	2977 (54)
2-3	566 (22)	254 (23)	302 (25)	129 (20)	1251 (23)
>3	494 (20)	266 (24)	255 (21)	98 (15)	1113 (20)
Uncertain	65 (3)	32 (3)	31 (2)	21 (3)	149 (3)
**Lymph node metastasis**	268 (11)	143 (13)	135 (11)	64 (10)	610 (11)
**Extrahepatic metastasis**	361 (14)	159 (15)	160 (13)	119 (18)	799 (15)
**Tumour thrombosis**	290 (12)	187 (17)	152 (12)	79 (12)	708 (13)
**Stage at diagnosis**					
Early	902 (36)	374 (34)	452 (37)	177 (27)	1905 (35)
Intermediate	530 (21)	226 (21)	262 (21)	120 (19)	1138 (21)
Advanced	324 (13)	153 (14)	153 (13)	61 (9)	691 (12)
Terminal	757 (30)	343 (31)	360 (29)	296 (45)	1756 (32)
**Comorbidities**					
Arterial hypertension	978 (39)	320 (29)	413 (34)	324 (50)	2035 (37)
Type 2 diabetes	1167 (46)	405 (37)	460 (37)	322 (49)	2354 (43)
Coronary artery disease	512 (20)	141 (13)	191 (16)	164 (25)	1008 (18)
Cerebrovascular disease	275 (11)	99 (9)	131 (11)	87 (13)	592 (11)
Chronic kidney disease	221 (9)	71 (6)	88 (7)	65 (10)	443 (8)
COPD	263 (10)	157 (14)	218 (18)	85 (13)	723 (13)
**Treatment**					

Transplantation	165 (6)	83 (8)	98 (8)	4 (1)	350 (6)
Resection	445 (18)	147 (13)	185 (15)	79 (12)	856 (15)
Ablation	365 (15)	189 (17)	215 (17)	90 (14)	859 (16)
Palliative	734 (29)	310 (28)	350 (29)	177 (27)	1571 (29)
Best supportive care	804 (32)	637 (34)	379 (31)	304 (46)	1854 (34)

COPD: chronic obstructive pulmonary disease; ECOG: Eastern Cooperative Oncology Group; MASLD: metabolic dysfunction-associated steatotic liver disease. [a] Percentage of patients with cirrhosis.

Table S5. Baseline characteristics of 5,490 patients diagnosed with hepatocellular carcinoma in Sweden between 2011 and 2021, stratified by educational level

	Educational level				
	Missing	Low	Medium	High	Total
	124 (2)	2080 (38)	2391 (44)	895 (16)	5490 (100)
**Male sex**	83 (67)	1565 (75)	1860 (78)	671 (75)	4179 (76)
**Median age**	73 (61-78)	72 (64-78)	68 (61-75)	69 (62-75)	70 (62-76)
**Country of birth**					
Nordic	34 (27)	1820 (88)	2113 (88)	727 (81)	4694 (85)
Non-Nordic	90 (73)	260 (12)	278 (12)	168 (19)	796 (15)
**Marital status**					
Married	68 (55)	920 (44)	1014 (42)	511 (57)	2513 (46)
Single	20 (16)	417 (20)	548 (23)	111 (13)	1096 (20)
Divorced/separated	14 (11)	438 (21)	585 (25)	190 (21)	1227 (22)
Widowed	22 (18)	305 (15)	244 (10)	83 (9)	654 (12)
**Household income**					
High	5 (4)	123 (6)	300 (13)	298 (33)	726 (13)
Medium	25 (20)	660 (32)	1127 (47)	406 (46)	2218 (41)
Low	94 (76)	1297 (62)	964 (40)	191 (21)	2546 (46)
**Neighbourhood deprivation**					
Q1 (least)	5 (4)	206 (10)	299 (12)	192 (21)	702 (13)
Q2	8 (6)	325 (16)	417 (17)	194 (22)	944 (17)
Q3	11 (9)	428 (20)	470 (20)	184 (21)	1093 (20)
Q4	27 (22)	494 (24)	565 (24)	154 (17)	1240 (23)
Q5 (most)	73 (59)	627 (30)	640 (27)	171 (19)	1498 (27)
**Aetiology**					
Hepatitis B	39 (31)	152 (8)	180 (8)	69 (8)	440 (8)
Hepatitis C	34 (27)	476 (23)	683 (29)	167 (18)	1360 (25)
Alcohol-related liver disease	10 (8)	317 (15)	487 (20)	216 (24)	1030 (19)
MASLD	18 (14)	645 (31)	578 (24)	226 (25)	1467 (27)
Other liver diseases	3 (2)	125 (6)	158 (7)	77 (9)	363 (6)
Cryptogenic cirrhosis	11 (9)	150 (7)	127 (5)	53 (6)	341 (6)
No diag. liver disease	9 (7)	215 (10)	178 (7)	87 (10)	489 (9)
**Liver cirrhosis**	102 (82)	1506 (72)	1870 (78)	686 (77)	4164 (76)
Child-Pugh ≤ 7[a]	79 (77)	1139 (76)	1446 (77)	521 (76)	3185 (76)
Child-Pugh >7[a]	23 (23)	367 (24)	424 (23)	165 (24)	979 (24)
**Diagnostic pathway**					
Surveillance	30 (24)	444 (21)	674 (28)	253 (28)	1401 (26)
Clinical symptoms	65 (52)	1141 (55)	1219 (51)	455 (51)	2880 (52)
Incidental finding	23 (19)	401 (19)	403 (17)	162 (18)	981 (18)
Missing	6 (5)	94 (5)	95 (4)	25 (3)	220 (4)
**ECOG PS**					
0	26 (21)	514 (25)	794 (33)	335 (37)	1669 (30)
1	36 (29)	637 (31)	742 (31)	276 (31)	1691 (31)
≥2	62 (50)	929 (44)	855 (36)	284 (32)	2130 (39)
**Tumour size (mm)**					
Median	50 (30-90)	50 (28-88)	40 (25-80)	40 (25-77)	45 (25-80)
≤20	17 (14)	314 (15)	440 (18)	171 (19)	942 (17)
21-29	20 (16)	276 (13)	380 (16)	146 (16)	822 (15)
≥30	87 (70)	1490 (72)	1571 (66)	578 (65)	3726 (68)
**Number of tumours**					
1	59 (48)	1141 (55)	1258 (53)	519 (58)	2977 (54)
2-3	31 (25)	466 (22)	562 (23)	192 (21)	1251 (23)
>3	30 (24)	418 (20)	504 (21)	161 (18)	1113 (20)
Uncertain	4 (3)	55 (3)	67 (3)	23 (3)	149 (3)
**Lymph node metastasis**	12 (10)	246 (12)	266 (11)	86 (10)	610 (11)
**Extrahepatic metastasis**	21 (17)	321 (15)	330 (14)	127 (14)	799 (15)
**Tumour thrombosis**	19 (15)	251 (12)	328 (14)	110 (12)	708 (13)
**Stage at diagnosis**					
Early	36 (29)	659 (32)	852 (36)	358 (40)	1905 (35)
Intermediate	25 (20)	416 (20)	512 (21)	185 (21)	1138 (21)
Advanced	18 (15)	209 (10)	342 (14)	122 (13)	691 (12)
Terminal	45 (36)	796 (38)	685 (29)	230 (26)	1756 (32)
**Comorbidities**					
Arterial hypertension	42 (34)	799 (38)	874 (37)	320 (36)	2035 (37)
Type 2 diabetes	45 (36)	944 (45)	1000 (42)	365 (41)	2354 (43)
Coronary artery disease	16 (13)	452 (22)	392 (16)	148 (17)	1008 (18)
Cerebrovascular disease	11 (9)	241 (12)	240 (10)	100 (11)	592 (11)
Chronic kidney disease	6 (5)	197 (9)	177 (7)	65 (7)	443 (8)
COPD	13 (10)	371 (15)	311 (13)	82 (9)	723 (13)
**Treatment**					

Transplantation	9 (7)	91 (4)	171 (7)	79 (9)	350 (6)
Resection	11 (9)	320 (15)	360 (15)	165 (18)	856 (15)
Ablation	18 (14)	289 (14)	404 (17)	148 (17)	859 (16)
Palliative	38 (31)	557 (27)	723 (30)	253 (28)	1571 (29)
Best supportive care	48 (39)	823 (40)	733 (31)	250 (28)	1854 (34)

COPD: chronic obstructive pulmonary disease; ECOG PS: Eastern Cooperative Oncology Group performance status; MASLD: metabolic dysfunction-associated steatotic liver disease. [a] Percentage of patients with cirrhosis.

Table S6. Baseline characteristics of 5,490 patients diagnosed with hepatocellular carcinoma in Sweden between 2011 and 2021, stratified by neighbourhood deprivation level

	Neighbourhood deprivation					
	**Q1 (least)**	**Q2**	**Q3**	**Q4**	**Q5 (most)**	**Total**
	702 (13)	944 (17)	1093 (20)	1240 (23)	1511 (27)	5490 (100)
**Male sex**	547 (78)	722 (76)	814 (74)	976 (79)	1120 (74)	4179 (76)
**Median age**	71 (65-76)	71 (63-76)	70 (63-77)	70 (62-77)	68 (61-76)	70 (62-76)
**Country of birth**						
Nordic	640 (91)	871 (92)	983 (90)	1087 (88)	1113 (74)	4694 (85)
Non-Nordic	62 (9)	73 (8)	110 (10)	153 (12)	398 (26)	796 (15)
**Marital status**						
Married	421 (60)	499 (53)	524 (48)	514 (42)	555 (37)	2513 (46)
Single	97 (14)	170 (18)	183 (17)	286 (23)	360 (24)	1096 (20)
Divorced/separated	111 (16)	181 (19)	232 (21)	286 (23)	417 (27)	1227 (22)
Widowed	73 (10)	94 (10)	154 (14)	154 (12)	179 (12)	654 (12)
**Household income**						
High	208 (30)	172 (18)	154 (14)	109 (9)	83 (5)	726 (13)
Medium	317 (45)	440 (47)	475 (44)	507 (41)	479 (32)	2218 (41)
Low	177 (25)	332 (35)	464 (42)	624 (50)	949 (63)	2546 (46)
**Educational level**						
High	192 (27)	194 (21)	184 (17)	154 (12)	171 (11)	895 (16)
Medium	299 (43)	417 (44)	470 (43)	565 (46)	640 (42)	2391 (44)
Low	206 (29)	325 (34)	428 (39)	494 (40)	627 (42)	2080 (38)
Unknown	5 (1)	8 (1)	11 (1)	27 (2)	73 (5)	124 (2)
**Aetiology**						
Hepatitis B	34 (5)	38 (4)	66 (6)	107 (9)	195 (13)	440 (8)
Hepatitis C	114 (16)	215 (23)	242 (22)	322 (26)	467 (31)	1360 (25)
Alcohol-related liver disease	173 (25)	209 (22)	221 (20)	200 (16)	227 (15)	1030 (19)
MASLD	206 (29)	259 (27)	302 (28)	335 (27)	365 (24)	1467 (27)
Other liver diseases	56 (8)	72 (8)	76 (7)	79 (6)	80 (5)	363 (6)
Cryptogenic cirrhosis	42 (6)	62 (7)	74 (7)	73 (6)	90 (6)	341 (6)
No diagnosed liver disease	77 (11)	89 (9)	112 (10)	124 (10)	87 (6)	489 (9)
**Liver cirrhosis**	543 (77)	700 (74)	813 (74)	914 (74)	1194 (79)	4164 (76)
Child-Pugh ≤ 7[a]	422 (78)	525 (75)	620 (76)	707 (77)	911 (76)	3185 (76)
Child-Pugh >7[a]	121 (22)	175 (25)	193 (24)	207 (23)	283 (24)	979 (24)
**Diagnostic pathway**						
Surveillance	179 (26)	214 (23)	283 (26)	290 (24)	435 (29)	1401 (26)
Clinical symptoms	383 (54)	523 (55)	554 (51)	657 (53)	732 (50)	2880 (52)
Incidental finding	118 (17)	172 (18)	200 (18)	241 (19)	258 (17)	981 (18)
Missing	22 (3)	34 (4)	56 (5)	52 (4)	56 (4)	220 (4)
**ECOG performance status**						
0	256 (37)	306 (32)	332 (30)	354 (29)	421 (28)	1669 (30)
1	213 (30)	302 (32)	328 (30)	390 (31)	458 (30)	1691 (31)
≥2	233 (33)	336 (36)	433 (40)	496 (40)	632 (42)	2130 (39)
**Tumour size (mm)**						
Median	44 (25-85)	46 (25-90)	45 (25-80)	45 (27-80)	42 (25-80)	45 (25-80)
≤20	126 (18)	174 (18)	181 (17)	190 (15)	268 (18)	942 (17)
21-30	105 (15)	135 (15)	158 (14)	182 (15)	242 (16)	822 (15)
≥30	468 (67)	635 (67)	754 (69)	868 (70)	1001 (66)	3726 (68)
**Number of tumours**						
1	377 (54)	531 (56)	602 (55)	653 (53)	814 (54)	2977 (54)
2-3	175 (25)	200 (21)	235 (22)	299 (24)	342 (22)	1251 (23)
>3	136 (19)	194 (21)	217 (20)	251 (20)	315 (21)	1113 (20)
Uncertain	14 (2)	19 (2)	39 (3)	37 (3)	40 (3)	149 (3)
**Regional metastasis**	77 (11)	113 (12)	112 (10)	143 (12)	165 (11)	610 (11)
**Extrahepatic metastasis**	110 (16)	125 (14)	155 (14)	177 (14)	229 (15)	799 (15)
**Tumour thrombosis**	83 (12)	128 (14)	135 (12)	161 (13)	201 (13)	708 (13)
**Stage at diagnosis**						
Early	271 (39)	341 (36)	369 (34)	424 (34)	500 (33)	1905 (35)
Intermediate	140 (20)	190 (20)	223 (20)	249 (20)	336 (22)	1138 (21)
Advanced	118 (17)	122 (13)	143 (13)	143 (12)	166 (11)	691 (12)
Terminal	173 (24)	291 (31)	359 (33)	424 (34)	509 (34)	1756 (32)
**Comorbidities**						
Arterial hypertension	289 (41)	360 (38)	418 (38)	444 (36)	524 (35)	2035 (37)
Type 2 diabetes	318 (45)	424 (45)	462 (42)	529 (43)	621 (41)	2354 (43)
Coronary artery disease	127 (18)	153 (16)	219 (20)	247 (20)	262 (17)	1008 (18)
Cerebrovascular disease	92 (13)	79 (8)	129 (12)	131 (11)	161 (11)	592 (11)
Chronic kidney disease	68 (10)	62 (7)	95 (9)	99 (8)	121 (8)	443 (8)
COPD	81 (11)	107 (11)	129 (12)	178 (14)	228 (15)	723 (13)

**Treatment**						
Transplantation	54 (8)	40 (4)	77 (7)	69 (6)	110 (7)	350 (6)
Resection	112 (16)	161 (17)	166 (15)	200 (16)	217 (14)	856 (15)
Ablation	127 (18)	166 (18)	160 (15)	182 (15)	224 (15)	859 (16)
Palliative	218 (31)	268 (28)	309 (28)	351 (28)	425 (28)	1571 (29)
Best supportive care	191 (27)	309 (33)	381 (35)	438 (35)	535 (36)	1854 (34)

COPD: chronic obstructive pulmonary disease; ECOG: Eastern Cooperative Oncology Group; MASLD: metabolic dysfunction-associated steatotic liver disease. [a] Percentage of patients with cirrhosis.

Table S7. Baseline characteristics of 5,490 patients diagnosed with hepatocellular carcinoma in Sweden between 2011 and 2021, stratified by cirrhosis status

	Liver cirrhosis		
	**Yes**	**No**	**Total**
	4164 (76)	1326 (24)	5490 (100)
**Male sex**	3251 (78)	928 (70)	4179 (76)
**Median age**	68 (61-74)	75 (69-81)	70 (62-76)
**Country of birth**			
Nordic	3493 (84)	1201 (91)	4694 (85)
Non-Nordic	671 (16)	125 (9)	796 (15)
**Marital status**			
Married	1843 (44)	670 (50)	2513 (46)
Single	914 (22)	182 (14)	1096 (20)
Divorced/separated	1005 (24)	222 (17)	1227 (22)
Widowed	402 (10)	252 (19)	654 (12)
**Household income**			
High	571 (13)	155 (12)	726 (13)
Medium	1635 (39)	583 (44)	2218 (41)
Low	1958 (47)	588 (44)	2546 (46)
**Educational level**			
High	686 (17)	209 (16)	895 (16)
Medium	1870 (45)	521 (39)	2391 (44)
Low	1506 (36)	574 (43)	2080 (38)
Unknown	102 (2)	22 (2)	124 (2)
**Neighbourhood deprivation**			
Q1 (least)	543 (13)	159 (12)	702 (13)
Q2	700 (17)	244 (18)	944 (17)
Q3	813 (19)	280 (21)	1093 (20)
Q4	976 (22)	326 (25)	1240 (23)
Q5 (most)	1194 (29)	317 (24)	1498 (27)
**Aetiology**			
Hepatitis B	412 (10)	28 (2)	440 (8)
Hepatitis C	1287 (31)	73 (6)	1360 (25)
Alcohol-related liver disease	987 (24)	43 (3)	1030 (19)
MASLD	829 (20)	638 (48)	1467 (27)
Other liver diseases	308 (7)	55 (4)	363 (6)
Cryptogenic cirrhosis	341 (8)	0	341 (6)
No diagnosed liver disease	0	489 (37)	489 (9)
**Diagnostic pathway**			
Surveillance	1387 (33)	14 (1)	1401 (26)
Clinical symptoms	1991 (48)	889 (67)	2880 (52)
Incidental finding	646 (16)	343 (26)	981 (18)
Missing	140 (3)	80 (6)	220 (4)
**ECOG performance status**			
0	1301 (31)	368 (28)	1669 (30)
1	1283 (31)	408 (31)	1691 (31)
≥2	1580 (38)	550 (41)	2130 (39)
**Tumour size (mm)**			
Median	38 (23-67)	75 (45-120)	45 (25-80)
≤20	837 (20)	105 (8)	942 (17)
21-29	739 (18)	83 (6)	822 (15)
≥30	2588 (62)	1138 (86)	3726 (68)
**Number of tumours**			
1	2106 (51)	871 (66)	2977 (54)
2-3	1052 (25)	199 (15)	1251 (23)
>3	898 (22)	215 (16)	1113 (20)
Uncertain	108 (2)	41 (3)	149 (3)
**Lymph node metastasis**	444 (11)	166 (13)	610 (11)
**Extrahepatic metastasis**	518 (12)	281 (21)	799 (15)
**Tumour thrombosis**	595 (14)	113 (9)	708 (13)
**Stage at diagnosis**			
Early	1460 (35)	445 (34)	1905 (35)
Intermediate	923 (22)	215 (16)	1138 (21)
Advanced	488 (12)	203 (15)	691 (12)
Terminal	1293 (31)	463 (35)	1756 (32)
**Comorbidities**			
Arterial hypertension	1470 (35)	565 (43)	2035 (37)
Type 2 diabetes	1812 (44)	542 (41)	2354 (43)
Coronary artery disease	663 (16)	345 (26)	1008 (18)
Cerebrovascular disease	409 (10)	183 (14)	592 (11)
Chronic kidney disease	316 (8)	129 (10)	443 (8)
COPD	544 (13)	179 (14)	723 (13)
**Treatment**			
Transplantation	347 (8)	3 (<1)	350 (6)
Resection	482 (12)	374 (28)	856 (15)

Ablation	797 (19)	62 (5)	859 (16)
Palliative	1167 (28)	404 (30)	1571 (29)
Best supportive care	1371 (33)	483 (36)	1854 (34)

COPD: chronic obstructive pulmonary disease; ECOG PS: Eastern Cooperative Oncology Group; MASLD: metabolic dysfunction-associated steatotic liver disease. [a] Percentage of patients with cirrhosis.

Table S8. Likelihood of HCC diagnosis during surveillance

	Univariable		Multivariable	
	**OR (95% CI)**	**P-value**	**aOR (95% CI)**	**P-value**
**Sex**				
Male	1.0 (ref)	-	1.0 (ref)	-
Female	1.16 (0.99-1.35)	0.058	1.31 (1.11-1.55)	0.001
**Age**	0.97 (0.96-0.98)	<0.001	0.98 (0.97-0.99)	<0.001
**Country of birth**				
Nordic	1.0 (ref)	-	1.0 (ref)	-
Non-Nordic	1.23 (1.04-1.46)	0.018	1.06 (0.87-1.30)	0.507
**Marital status**				
Married	1.0 (ref)	-	1.0 (ref)	-
Single	1.11 (0.94-1.31)	0.230	0.98 (0.81-1.19)	0.155
Divorced/separated	1.22 (1.04-1.44)	0.014	1.16 (0.98-1.39)	0.818
Widowed	0.58 (0.45-0.75)	<0.001	0.72 (0.57-0.95)	0.020
**Household income**				
High	1.0 (ref)	-	1.0 (ref)	-
Medium	0.90 (0.74-1.10)	0.291	0.95 (0.77-1.18)	0.503
Low	0.70 (0.58-0.85)	<0.001	0.63 (0.50-0.80)	<0.001
**Educational level**				
High	1.0 (ref)	-	1.0 (ref)	-
Medium	0.96 (0.80-1.15)	0.631	1.00 (0.82-1.22)	0.792
Low	0.72 (0.59-0.87)	0.001	0.88 (0.65-1.08)	0.255
**Neighbourhood deprivation**				
Q1 (least)	1.0 (ref)	-	1.0 (ref)	-
Q2	0.88 (0.70-1.13)	0.320	0.84 (0.65-1.08)	0.120
Q3	1.07 (0.85-1.35)	0.558	1.06 (0.83-1.35)	0.770
Q4	0.95 (0.76-1.19)	0.646	0.92 (0.72-1.17)	0.395
Q5 (most)	1.15 (0.93-1.43)	0.191	1.14 (0.90-1.45)	0.420
**Period**				
2011-2024	1.0 (ref)	-	1.0 (ref)	-
2015-2019	1.59 (1.36-1.85)	<0.001	1.84 (1.56-2.16)	<0.001
2020-2021	1.61 (1.33-1.94)	<0.001	1.96 (1.60-2.41)	<0.001
**Aetiology**				
Hepatitis C	1.0 (ref)	-	1.0 (ref)	-
Hepatitis B	0.80 (0.64-1.00)	0.054	0.72 (0.57-0.94)	0.014
Alcohol-related liver disease	0.58 (0.48-0.68)	<0.001	0.56 (0.46-0.68)	<0.001
MASLD	0.29 (0.23-0.34)	<0.001	0.30 (0.23-0.37)	<0.001
Other liver diseases	0.78 (0.61-1.00)	0.056	0.70 (0.53-0.93)	0.013
Cryptogenic cirrhosis	0.23 (0.17-0.32)	<0.001	0.24 (0.17-0.34)	<0.001

CI: confidence interval; HCC: hepatocellular carcinoma; MASLD: metabolic dysfunction-associated steatotic liver disease, OR: odds ratio. Results from univariable and multivariable logistic regression models. The multivariable model, which included all variables in this table, was statistically significant compared to the null model (Chi square (21) = 367.63, $p < 0.001$), and correctly classified 68% of cases.

Table S9. Likelihood of HCC diagnosis at early-stage

	Univariable		Multivariable	
	**OR (95% CI)**	**P-value**	**aOR (95% CI)**	**P-value**
**Sex**				
Male	1.0 (ref)	-	1.0 (ref)	-
Female	1.18 (1.04-1.34)	0.011	1.33 (1.14-1.54)	<0.001
**Age**	0.97 (0.96-0.98)	<0.001	0.98 (0.97-0.99)	<0.001
**Country of birth**				
Nordic	1.0 (ref)	-	1.0 (ref)	-
Non-Nordic	1.34 (1.15-1.57)	<0.001	1.29 (1.07-1.57)	0.008
**Marital status**				
Married	1.0 (ref)	-	1.0 (ref)	-
Single	0.93 (0.78-1.07)	0.307	0.85 (0.72-1.02)	0.082
Divorced/separated	1.04 (0.90-1.20)	0.573	1.01 (0.87-1.19)	0.795
Widowed	0.66 (0.55-0.80)	<0.001	0.93 (0.75-1.16)	0.532
**Household income**				
High	1.0 (ref)	-	1.0 (ref)	-
Medium	0.88 (0.74-1.04)	0.144	1.04 (0.86-1.25)	0.692
Low	0.52 (0.44-0.62)	<0.001	0.58 (0.51-0.67)	<0.001
**Educational level**				
High	1.0 (ref)	-	1.0 (ref)	-
Medium	0.83 (0.71-0.97)	0.021	0.92 (0.78-1.10)	0.967
Low	0.70 (0.59-0.82)	<0.001	1.01 (0.78-1.22)	0.296
**Neighbourhood deprivation**				
Q1 (least)	1.0 (ref)	-	1.0 (ref)	-
Q2	0.90 (0.74-1.10)	0.303	0.90 (0.72-1.11)	0.120
Q3	0.81 (0.67-0.99)	0.037	1.01 (0.67-1.02)	0.770
Q4	0.83 (0.68-1.00)	0.052	0.89 (0.72-1.09)	0,395
Q5 (most)	0.79 (0.65-0.95)	0.011	0.81 (0.66-1.00)	0.420
**Period**				
2011-2024	1.0 (ref)	-	1.0 (ref)	-
2015-2019	1.28 (1.13-1.46)	<0.001	1.39 (1.21-1.60)	<0.001
2020-2021	1.26 (1.08-1.49)	0.005	1.43 (1.20-1.70)	<0.001
**Cirrhosis status**				
Compensated	1.0 (ref)	-	1.0 (ref)	-
Decompensated	0.32 (0.26-0.38)	<0.001	0.29 (0.24-0.35)	<0.001
No cirrhosis	0.74 (0.65-0.85)	<0.001	1.03 (0.86-1.24)	0.742
**Aetiology**				
Hepatitis C	1.0 (ref)	-	1.0 (ref)	-
Hepatitis B	1.04 (0.84-1.30)	0.688	0.86 (0.67-1.11)	0.252
Alcohol-related liver disease	0.78 (0.66-0.92)	0.003	0.88 (0.73-1.05)	0.166
MASLD	0.63 (0.54-0.73)	<0.001	0.70 (0.57-0.85)	<0.001
Other liver diseases	1.11 (0.88-1.40)	0.387	1.13 (0.87-1.47)	0.353
Cryptogenic cirrhosis	0.31 (0.23-0.42)	<0.001	0.34 (0.25-0.48)	<0.001
No liver disease	0.65 (0.52-0.81)	<0.001	0.59 (0.44-0.80)	0.001

CI: confidence interval; HCC: hepatocellular carcinoma; MASLD: metabolic dysfunction-associated steatotic liver disease, OR: odds ratio. Results from univariable and multivariable logistic regression models. The multivariable model, which included all variables in this table, was statistically significant compared to the null model (Chi square (24) = 327.90, $p < 0.001$), and correctly classified 66% of cases.

Table S10. Likelihood of receiving curative treatment after HCC diagnosis

	Univariable		Multivariable	
	OR (95% CI)	P-value	aOR (95% CI)	P-value
**Sex**				
Male	1.0 (ref)	-	1.0 (ref)	-
Female	1.14 (1.01-1.30)	0.037	1.14 (0.94-1.38)	0.196
**Age**	0.96 (0.95-0.97)	<0.001	0.96 (0.95-0.97)	<0.001
**Country of birth**				
Nordic	1.0 (ref)	-	1.0 (ref)	-
Non-Nordic	1.36 (1.17-1.59)	<0.001	1.06 (0.82-1.36)	0.663
**Marital status**				
Married	1.0 (ref)	-	1.0 (ref)	-
Single	0.98 (0.84-1.13)	0.747	0.96 (0.76-1.21)	0.727
Divorced/separated	1.08 (0.94-1.24)	0.293	1.18 (0.96-1.45)	0.117
Widowed	0.57 (0.47-0.69)	<0.001	0.85 (0.65-1.13)	0.264
**Household income**				
High	1.0 (ref)	-	1.0 (ref)	-
Medium	0.82 (0.69-0.97)	0.019	0.88 (0.69-1.12)	0.307
Low	0.49 (0.41-0.57)	<0.001	0.65 (0.50-0.85)	<0.001
**Educational level**				
High	1.0 (ref)	-	1.0 (ref)	-
Medium	0.82 (0.71-0.96)	0.015	0.94 (0.75-1.18)	0.592
Low	0.65 (0.55-0.76)	<0.001	0.97 (0.77-1.23)	0.820
**Neighbourhood deprivation**				
Q1 (least)	1.0 (ref)	-	1.0 (ref)	-
Q2	0.89 (0.72-1.08)	0.242	0.86 (0.65-1.13)	0.279
Q3	0.82 (0.67-0.99)	0.039	0.81 (0.62-1.07)	0.144
Q4	0.80 (0.66-0.96)	0.020	0.87 (0.66-1.14)	0.305
Q5 (most)	0.80 (0.67-0.96)	0.018	0.82 (0.62-1.08)	0.152
**Period**				
2011-2024	1.0 (ref)	-	1.0 (ref)	-
2015-2019	1.30 (1.14-1.47)	<0.001	1.28 (0.99-1.53)	0.054
2020-2021	1.39 (1.18-1.62)	<0.001	1.10 (0.87-1.38)	0.423
**Cirrhosis status**				
Compensated	1.0 (ref)	-	1.0 (ref)	-
Decompensated	0.25 (0.21-0.30)	<0.001	0.33 (0.26-0.43)	<0.001
No cirrhosis	0.59 (0.51-0.67)	<0.001	1.18 (0.92-1.52)	0.189
**Aetiology**				
Hepatitis C	1.0 (ref)	-	1.0 (ref)	-
Hepatitis B	1.13 (0.91-1.40)	0.260	1.27 (0.91-1.77)	0.161
Alcohol-related liver disease	0.85 (0.72-1.00)	0.053	1.10 (0.86-1.41)	0.433
MASLD	0.59 (0.51-0.69)	<0.001	1.18 (0.88-1.57)	0.259
Other liver diseases	1.24 (0.98-1.56)	0.072	1.48 (1.04-2.11)	0.028
Cryptogenic cirrhosis	0.30 (0.22-0.40)	<0.001	0.86 (0.57-1.29)	0.460
No liver disease	0.57 (0.46-0.71)	<0.001	1.20 (0.81-1.77)	0.365
**ECOG performance status**				
0	1.0 (ref)	-	1.0 (ref)	-
1	0.32 (0.27-0.36)	<0.001	0.41 (0.34-0.49)	<0.001
≥2	0.08 (0.07-0.10)	<0.001	0.16 (0.12-0.19)	<0.001
**Tumour size (mm)**				
≤20	1.0 (ref)	-	1.0 (ref)	-
21-29	0.78 (0.64-0.96)	0.017	0.83 (0.65-1.07)	0.150
≥30	0.13 (0.11-0.15)	<0.001	0.18 (0.14-0.22)	<0.001
**Number of tumours**				
1	1.0 (ref)	-	1.0 (ref)	-
2-3	0.72 (0.63-0.83)	<0.001	0.57 (0.48-0.68)	<0.001
>3	0.09 (0.08-0.12)	<0.001	0.12 (0.09-0.16)	<0.001
**Lymph node metastasis**	0.13 (0.10-0.18)	<0.001	0.44 (0.30-0.65)	<0.001
**Extrahepatic metastasis**	0.04 (0.03-0.06)	<0.001	0.08 (0.05-0.13)	<0.001
**Tumour thrombosis**	0.04 (0.03-0.06)	<0.011	0.08 (0.05-0.13)	<0.001
**Comorbidities**				
Arterial hypertension	0.66 (0.59-0.74)	<0.001	0.84 (0.70-0.99)	0.049
Type 2 diabetes	1.00 (0.90-1.12)	0.930	1.16 (0.96-1.40)	0.124
Coronary artery disease	0.76 (0.65-0.88)	<0.001	0.92 (0.75-1.13)	0.432
Cerebrovascular disease	0.72 (0.60-0.87)	0.001	1.18 (0.90-1.55)	0.234
Chronic kidney disease	1.02 (0.84-1.25)	0.789	0.96 (0.73-1.21)	0.753
COPD	0.86 (0.73-1.01)	0.071	0.94 (0.73-1.21)	0.627

CI: confidence interval; COPD: Chronic obstructive pulmonary disease; HCC: hepatocellular carcinoma; MASLD: metabolic dysfunction-associated steatotic liver disease, OR: odds ratio. Results from univariable and multivariable logistic regression models. The multivariable model, which included all variables in this table, was statistically significant compared to the null model (Chi square (42) = 2914.06, $p < 0.001$), and correctly classified 89% of cases.

Table S11. Mortality risk after HCC diagnosis

	Univariable		Multivariable 1		Multivariable 2	
	**HR (95% CI)**	**P-value**	**aHR (95% CI)**	**P-value**	**aHR (95% CI)**	**P-value**
**Sex**						
Male	1.0 (ref)	-	1.0 (ref)	-	1.0 (ref)	-
Female	0.94 (0.88-1.01)	0.091	0.94 (0.87-1.02)	0.092	0.93 (0.86-1.01)	0.092
**Age**	1.02 (1.02-1.03)	<0.001	1.02 (1.01-1.02)	<0.001	1.02 (1.01-1.02)	<0.001
**Country of birth**						
Nordic	1.0 (ref)	-	1.0 (ref)	-	1.0 (ref)	-
Non-Nordic	0.77 (0.71-0.84)	<0.001	0.89 (0.80-0.99)	0.045	0.90 (0.81-0.99)	0.048
**Marital status**						
Married	1.0 (ref)	-	1.0 (ref)	-	1.0 (ref)	-
Single	1.02 (0.94-1.11)	0.596	1.06 (0.97-1.16)	0.204	1.06 (0.97-1.16)	0.197
Divorced/separated	0.96 (0.89-1.03)	0.272	0.98 (0.90-1.06)	0.651	0.98 (0.90-1.06)	0.669
Widowed	1.34 (1.21-1.47)	<0.001	1.04 (0.93-1.15)	0.496	1.04 (0.94-1.15)	0.476
**Household income**						
High	1.0 (ref)	-	1.0 (ref)	-	1.0 (ref)	-
Medium	1.11 (1.01-1.23)	0.033	0.99 (0.90-1.10)	0.685	1.09 (0.96-1.21)	0.154
Low	1.52 (1.38-1.67)	<0.001	1.29 (1.15-1.45)	<0.001	1.32 (1.18-1.53)	<0.001
**Educational level**						
High	1.0 (ref)	-	1.0 (ref)	-	1.0 (ref)	-
Medium	1.08 (0.99-1.18)	0.083	0.94 (0.86-1.04)	0.374	0.96 (0.87-1.05)	0.384
Low	1.29 (1.18-1.41)	<0.001	0.99 (0.90-1.09)	0.810	1.00 (0.92-1.11)	0.854
**Neighbourhood deprivation**						
Q1 (least)	1.0 (ref)	-	1.0 (ref)	-	1.0 (ref)	-
Q2	1.01 (0.91-1.13)	0.859	1.00 (0.90-1.12)	0.938	1.00 (0.90-1.13)	0.876
Q3	1.07 (0.96-1.19)	0.214	1.09 (0.97-1.21)	0.170	1.08 (0.97-1.21)	0.158
Q4	1.09 (0.98-1.21)	0.102	1.02 (0.92-1.14)	0.748	1.02 (0.91-1.13)	0.739
Q5 (most)	1.05 (0.95-1.16)	0.310	1.03 (0.92-1.15)	0.627	1.03 (0.92-1.15)	0.592
**Period**						
2011-2024	1.0 (ref)	-	1.0 (ref)	-	1.0 (ref)	-
2015-2019	0.90 (0.84-0.96)	0.002	0.90 (0.84-0.96)	0.002	0.90 (0.84-0.96)	0.002
2020-2021	0.85 (0.77-0.93)	<0.001	0.89 (0.81-0.98)	0.016	0.89 (0.81-0.98)	0.017
**Cirrhosis status**						
Compensated	1.0 (ref)	-	1.0 (ref)	-	1.0 (ref)	-
Decompensated	2.15 (1.99-2.33)	<0.001	1.70 (1.56-1.85)	<0.001	1.70 (1.56-1.85)	<0.001
No cirrhosis	1.27 (1.18-1.36)	<0.001	0.87 (0.78-0.96)	0.006	0.87 (0.78-0.96)	0.006
**Aetiology**						
Hepatitis C	1.0 (ref)	-	1.0 (ref)	-	1.0 (ref)	-
Hepatitis B	0.97 (0.85-1.10)	0.620	0.97 (0.85-1.12)	0.709	0.98 (0.85-1.12)	0.734
Alcohol-related liver disease	1.26 (1.15-1.38)	<0.001	1.12 (1.01-1.24)	0.023	1.12 (1.01-1.24)	0.027
MASLD	1.45 (1.33-1.58)	<0.001	1.10 (0.98-1.24)	0.088	1.10 (0.98-1.24)	0.093
Other liver diseases	0.98 (0.86-1.13)	0.787	0.99 (0.86-1.16)	0.974	0.99 (0.85-1.16)	0.933
Cryptogenic cirrhosis	1.99 (1.75-2.25)	<0.001	1.13 (0.98-1.31)	0.083	1.13 (0.98-1.30)	0.087
No liver disease	1.38 (1.23-1.55)	<0.001	1.13 (0.96-1.33)	0.133	1.13 (0.96-1.33)	0.132
**ECOG performance status**						
0	1.0 (ref)	-	1.0 (ref)	-	1.0 (ref)	-
1	1.75 (1.61-1.89)	<0.001	1.38 (1.27-1.50)	<0.001	1.38 (1.27-1.50)	<0.001
≥2	3.61 (3.35-3.90)	<0.001	2.21 (2.03-2.41)	<0.001	2.22 (2.03-2.42)	<0.001
**Tumour size (mm)**						
≤20	1.0 (ref)	-	1.0 (ref)	-	1.0 (ref)	-
21-29	1.19 (1.06-1.34)	0.003	1.14 (1.01-1.28)	0.034	1.13 (1.01-1.28)	0.035
≥30	2.47 (2.26-2.70)	<0.001	1.70 (1.54-1.88)	<0.001	1.70 (1.54-1.87)	<0.001
**Number of tumours**						
1	1.0 (ref)	-	1.0 (ref)	-	1.0 (ref)	-
2-3	1.14 (1.06-1.23)	<0.001	1.16 (1.08-1.26)	<0.001	1.16 (1.08-1.26)	<0.001
>3	2.67 (2.48-2.88)	<0.001	1.77 (1.63-1.93)	<0.001	1.77 (1.63-1.93)	<0.001
**Lymph node metastasis**	2.63 (2.40-2.88)	<0.001	1.43 (1.29-1.58)	<0.001	1.43 (1.30-1.58)	<0.001
**Extrahepatic metastasis**	3.53 (3.25-3.83)	<0.001	1.95 (1.78-2.14)	<0.001	1.94 (1.78-2.14)	<0.001
**Tumour thrombosis**	3.77 (3.47-4.11)	<0.011	2.32 (2.11-2.54)	<0.001	2.31 (2.10-2.54)	<0.001
**Comorbidities**						
Arterial hypertension	1.31 (1.23-1.39)	<0.001	1.09 (1.01-1.16)	0.018	1.09 (1.02-1.17)	0.017
Type 2 diabetes	1.05 (0.99-1.12)	0.060	0.96 (0.89-1.04)	0.361	0.97 (0.89-1.04)	0.371
Coronary artery disease	1.24 (1.15-1.33)	<0.001	1.07 (0.98-1.16)	0.121	1.06 (0.98-1.15)	0.133
Cerebrovascular disease	1.26 (1.15-1.39)	<0.001	1.14 (1.02-1.27)	0.025	1.13 (1.01-1.27)	0.027
Chronic kidney disease	1.18 (1.06-1.31)	0.002	1.07 (0.98-1.17)	0.135	1.07 (0.98-1.17)	0.143
COPD	1.17 (1.07-1.28)	<0.001	1.05 (0.95-1.16)	0.308	1.05 (0.95-1.16)	0.303

CI: confidence interval; COPD: Chronic obstructive pulmonary disease; HCC: hepatocellular carcinoma; MASLD: metabolic dysfunction-associated steatotic liver disease, OR: odds ratio. Results from univariable and multivariable Cox regression models. The multivariable model 1 included all variables in this table, while for multivariable model 2 household income was also included as a time-dependent variable having aHR of 1.03 (95% CI: 1.01-1.06).

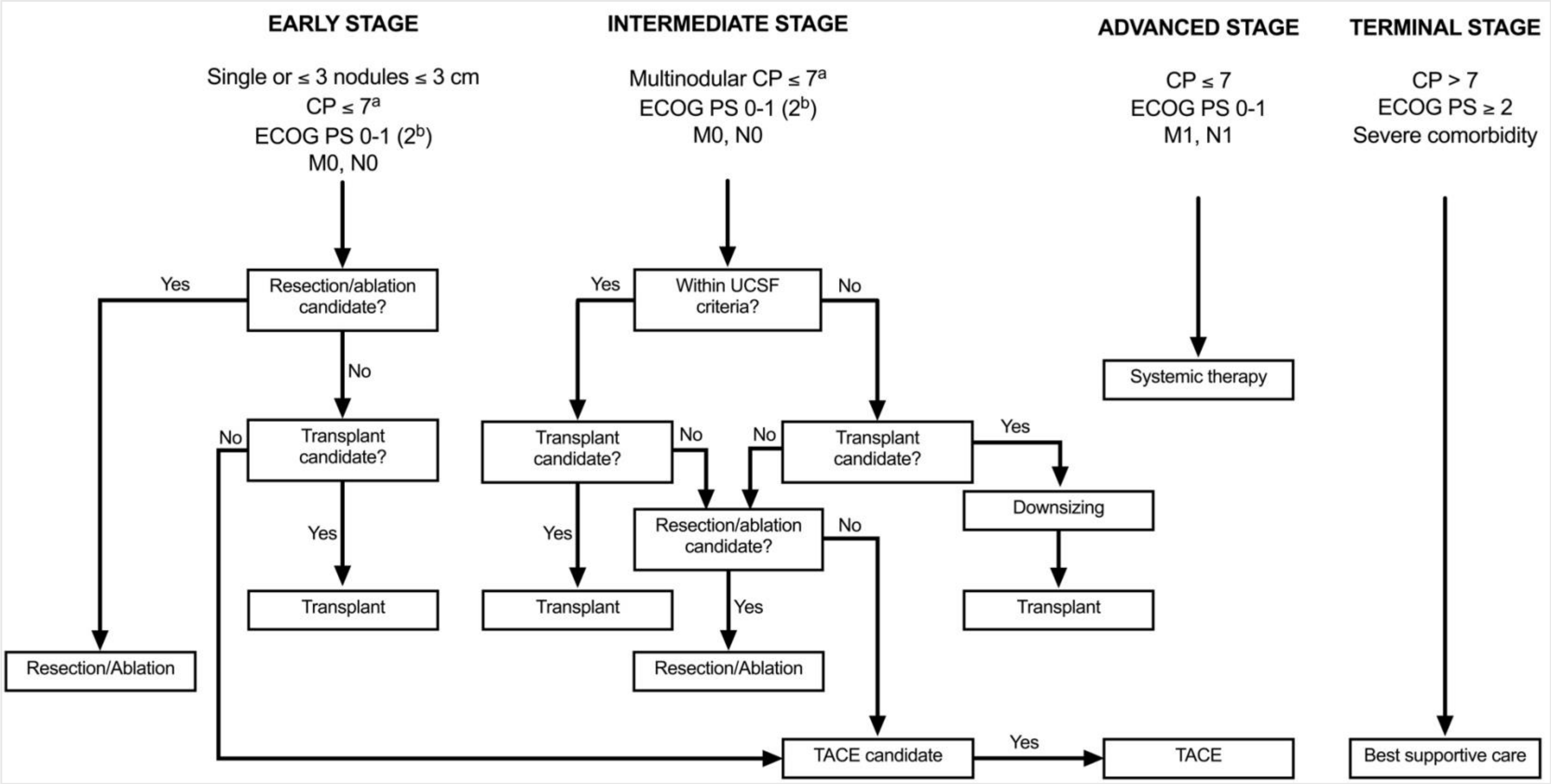

Figure S1. Swedish treatment algorithm for hepatocellular carcinoma. CP: Child-Pugh score; ECOG PS: Eastern Cooperative Oncology Group performance status; M: distant metastasis; N: regional lymph node metastasis; TACE: transarterial chemoembolisation; UCSF: University of California San Francisco. [a] CP non-relevant for liver transplant candidates. [b] Some patients with ECOG PS = 2 might become candidates for treatment with curative intent after individual evaluation at multidisciplinary team cancer conferences.

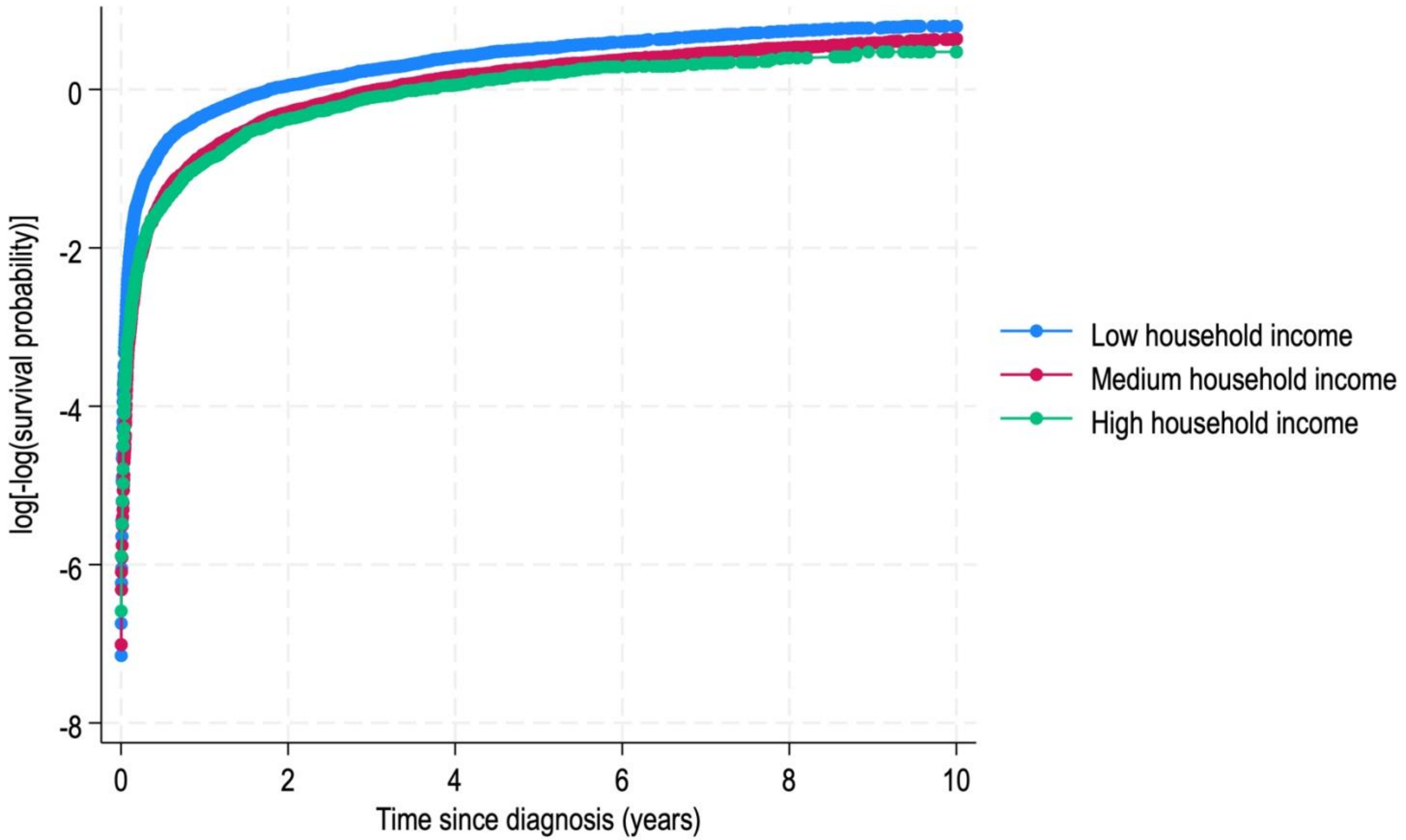

Figure S2. Log-log(survival) for household income.
